# Apoptotic Bodies Derived from Fibroblast‐Like Cells in Subcutaneous Connective Tissue Inhibit Ferroptosis in Ischaemic Flaps via the miR‐339‐5p/KEAP1/Nrf2 Axis

**DOI:** 10.1002/advs.202307238

**Published:** 2024-04-19

**Authors:** Gaoxiang Yu, Yijie Chen, Ningning Yang, Haojie Zhang, Xuzi Zhang, Yibo Geng, Jiayi Zhao, Zhuliu Chen, Chengji Dong, Lidan Lin, Jianjun Qi, Xuanlong Zhang, Xiaoqiong Jiang, Weiyang Gao, Yuepiao Cai, Xiangyang Wang, Jian Ding, Jian Xiao, Kailiang Zhou

**Affiliations:** ^1^ Department of Orthopaedics The Second Affiliated Hospital and Yuying Children's Hospital of Wenzhou Medical University Wenzhou 325027 China; ^2^ Zhejiang Provincial Key Laboratory of Orthopaedics Wenzhou 325027 China; ^3^ The Second Clinical Medical College of Wenzhou Medical University Wenzhou 325027 China; ^4^ Department of Obstetrics and Gynecology The Second Affiliated Hospital of Wenzhou Medical University Wenzhou Zhejiang 325027 China; ^5^ School of Basic Medical Sciences Institute of Hypoxia Research Wenzhou Medical University Wenzhou 325035 China; ^6^ Department of Clinical Laboratory The First Affiliated Hospital of Wannan Medical College Wuhu Anhui 241001 China; ^7^ Molecular Pharmacology Research Center School of Pharmaceutical Science Wenzhou Medical University Wenzhou 325000 China

**Keywords:** apoptotic bodies, ferroptosis, ischaemic flaps, KEAP1‐Nrf2, miR‐339‐5p

## Abstract

Preventing and treating avascular necrosis at the distal end of the flaps are critical to surgery success, but current treatments are not ideal. A recent study shows that apoptotic bodies (ABs) generated near the site of apoptosis can be taken up and promote cell proliferation. The study reveals that ABs derived from fibroblast‐like cells in the subcutaneous connective tissue (FSCT cells) of skin flaps promoted ischaemic flap survival. It is also found that ABs inhibited cell death and oxidative stress and promoted M1‐to‐M2 polarization in macrophages. Transcriptome sequencing and protein level testing demonstrated that ABs promoted ischaemic flap survival in endothelial cells and macrophages by inhibiting ferroptosis via the KEAP1‐Nrf2 axis. Furthermore, microRNA (miR) sequencing data and in vitro and in vivo experiments demonstrated that ABs inhibited KEAP1 by delivering miR‐339‐5p to exert therapeutic effects. In conclusion, FSCT cell‐derived ABs inhibited ferroptosis, promoted the macrophage M1‐to‐M2 transition via the miR‐339‐5p/KEAP1/Nrf2 axis and promoted ischaemic flap survival. These results provide a potential therapeutic strategy to promote ischaemic flap survival by administering ABs.

## Graphical abstract

Preventing and treating avascular necrosis at the distal end of the flap are critical to surgery success, but current treatments are not ideal. The present study reveals subcutaneous connective tissue cells‐derived ABs inhibited ferroptosis via the miR‐339‐5p/KEAP1/Nrf2 axis and promoted ischaemic flap survival. The finding provides a potential therapeutic strategy to promote ischaemic flap survival by administering ABs.

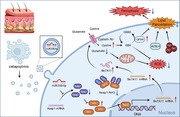

## 1 Introduction

Skin flap transplantation is widely used in large‐area wound repair, plastic surgery, and routine applications.^[^ ^1^ ^]^ Unstable factors associated with ischaemic necrosis are present at the distal end of the flap because the blood supply is lacking, which markedly restricts the clinical application of skin flap transplantation.^[^ ^2^ ^]^ Consequently, identifying a new strategy to significantly improve the survival rates of distal ischaemic flaps is of major clinical and scientific importance. Random‐pattern skin flap (FLAP) models are often used in studies of ischaemic flaps due to their convenience and ease of use.^[^ ^3^ ^]^ The known mechanisms underlying ischaemic flaps are mainly the lack of angiogenesis, the accumulation of oxygen free radicals, the infiltration of inflammatory cells, and other harmful events.^[^ ^4^ ^]^ Promoting flap survival mostly relies on enhancing vascular proliferation and minimizing inflammation^[^ ^5^ ^]^; therefore, we focused on the pivotal roles of endothelial cells and macrophages. However, the treatment methods for reducing distal ischaemic necrosis are not ideal.

Extracellular vesicles (EVs), which include exosomes, microvesicles, retrovirus‐like particles (RLPs), and apoptotic bodies (ABs), are thought to act as carriers responsible for intercellular signal exchange and signal transduction.^[^ ^6^ ^]^ During normal cellular processes, exosomes, microvesicles, and RLPs are secreted, and during apoptosis, only ABs are formed.^[^ ^7^ ^]^ To date, accumulating evidence suggests that ABs produced via apoptosis are closely related to tissue regeneration. A skin wound healing model showed that mesenchymal stem cell (MSC) transplantation was key to the production of ABs because many MSCs undergo apoptosis.^[^ ^8^ ^]^ In zebrafish, ABs produced via epithelial cell apoptosis are phagocytosed by surrounding normal endothelial cells to promote their proliferation.^[^ ^9^ ^]^ This finding suggests that ABs may exert therapeutic effects on ischaemic skin flaps. Compared to other sources, autologous cells are safer and more reliable sources for the clinical use of EVs due to the potential risk of immune rejection posed by proteins.^[^ ^10^ ^]^ Furthermore, their accessibility and ability to be produced in large quantities make them more suitable for clinical translation than stem cells.^[^ ^11^ ^]^ Therefore, L‐wnt‐3A cells (fibroblast‐like cells from subcutaneous connective tissue, FSCT cells) were chosen as the blast cells for ABs extraction.

Ferroptosis is a form of programmed cell death (PCD) dependent on iron and driven by lipid peroxide overload on cell membranes.^[^ ^12^ ^]^ The main defence system includes cystine/glutamate antiporters (system Xc‐, which is composed of the light chain SLC7A11 (xCT) and the heavy chain SLC3A2 (4F2hc)), which are activated during ferroptosis, resulting in glutamate accumulation and cystine reduction after injury.^[^ ^13^ ^]^ Decreased cystine levels lead to reduced recombinant thioredoxin reductase 1 (Txnrd1)‐induced glutathione (GSH) production and inhibit recombinant glutathione peroxidase 4 (GPX4) activity.^[^ ^14^ ^]^ Excess reactive oxygen species (ROS) accumulate to cause lipid peroxidation of the cell membrane, which is affected by acyl‐CoA synthetase long‐chain family member 4 (ACSL4) and Fe^2+^, ultimately leading to ferroptosis.^[^ ^15^ ^]^ The literature indicates that ferroptosis is closely linked to macrophage polarization.^[^ ^16^ ^]^ Ferroptosis induces the production of damage‐associated molecular patterns (DAMPs), which results in the secretion of endogenous danger signals, including 8‐OHdG and HMGB‐1.^[^ ^17^ ^]^ This triggers M1 macrophage polarization, and these cells further release proinflammatory cytokines (such as TNF‐*α* and IFN‐*β*) and promote ROS accumulation, which potentiates ferroptosis.^[^ ^18^ ^]^ The Kelch‐like ECH‐associated protein 1‐NF‐E2‐related factor 2 (KEAP1‐Nrf2) axis is a primary defence against exogenous and endogenous oxidative and electrophilic stressors.^[^ ^19^ ^]^ Nrf2 is a transcription factor that regulates the expression of antioxidant‐ and detoxification‐related genes.^[^ ^20^ ^]^ Numerous studies have demonstrated that EVs suppress ferroptosis by targeting Nrf2, which increases the expression of the antioxidant gene SLC7A11.^[^ ^21^ ^]^ Pre‐experimental transcriptome results indicate that the KEAP1‐Nrf2 pathway changes under the action of ABs. These findings suggest that the KEAP‐Nrf2 axis in ischaemic flaps may regulate levels of ferroptosis and macrophage polarization.

MicroRNAs (miRs), which are a class of small noncoding RNAs, recognize homologous sequences and interfere with transcriptional, translational or epigenetic processes to regulate gene expression.^[^ ^22^ ^]^ Recently, many studies have reported that miRs play a major role in disease processes.^[^ ^23^ ^]^ Circulating ABs effectively promote the self‐renewal and osteogenic/adipogenic differentiation of bone marrow MSCs by delivering miR‐328‐3p.^[^ ^24^ ^]^ miR‐21‐5p carried by ABs from adipose‐derived MSCs induces M2 macrophage polarization and promotes skin wound healing.^[^ ^25^ ^]^ In a study of EVs, the delivery of miRs was suggested to be a key factor in their therapeutic effect.^[^ ^26^ ^]^ ABs are a type of EV, and ABs are the most enriched in miRs among all EV subtypes.^[^ ^27^ ^]^ To clarify which miR plays a key role in FSCT cell‐derived ABs, miR sequencing was performed on samples from the Control and ABs flap group, as well as ABs themselves. According to the sequencing data, FSCT cell‐derived ABs carried the same 30 miRs whose expression was increased in tissues. Furthermore, we examined whether these miRs targeted KEAP1. By comparing the miRs targeting KEAP1 in the TargetScan and Diana databases with these 30 miRs, we identified miR‐339‐5p.

This study used FSCT cell‐derived ABs to promote the survival of distal ischaemic flaps. ABs treatment inhibited oxidative stress and cell death, promoted the differentiation of macrophages from the M1 to the M2 phenotype, inhibited KEAP1 expression and promoted Nrf2 nuclear translocation to inhibit ferroptosis in endothelial cells and macrophages. Interestingly, miR‐339‐5p, which was identified by miR sequencing, was present in ABs and carried into flap tissue cells, and miR‐339‐5p achieved targeted inhibition of KEAP1 to mediate these therapeutic effects. We also demonstrated the feasibility of these strategies in vitro. Therefore, this study examined how ABs contribute to the survival of ischaemic flaps by carrying miR‐339‐5p.

## 2 Results

### 2.1 Characterization of FSCT Cell‐Derived ABs

**Figure 1:**
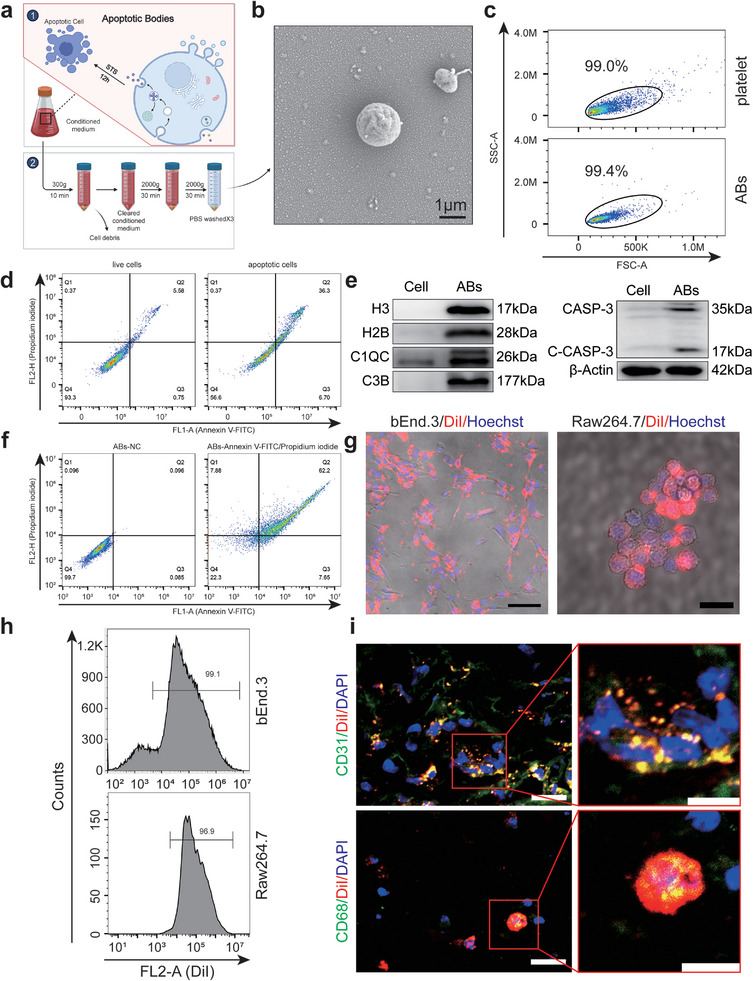
Isolation and characterization of FSCT cell‐derived ABs. a) Graphical illustration of the ABs isolation procedure. b) AB morphology under SEM. c) FSC/SSC analysis of ABs. Frames were used as size markers (platelets, 1–4 µm) to gate the ABs. d) FCM of live and apoptotic FSCT cells (annexin V/PI). e) WB analysis of C1QC, H3, C1QC, C3B, H2B, CASP‐3, cl‐CASP‐3 and *β*‐actin expression in the indicated groups. f) FCM analysis of ABs treated with NC or Annexin V/PI. g) Uptake of ABs labeled with the red fluorescent die DiI by bEnd.3 (scale bar: 100 µm) and Raw264.7 cells (scale bar: 25 µm). h) FCM analysis of the percentage of DiI‐positive bEnd.3 and Raw264.7 cells after treatment with DiI‐labelled ABs. i) Frozen sections of DiI‐labelled ABs‐treated skin tissues on POD3 were stained for CD31 or CD68. Scale bars: left, 20 µm; right, 10 µm.

To determine whether the increased number of ABs produced by adjacent tissue cells in ischaemic flaps could promote flap survival, FSCT cells were used to produce ABs. Flow cytometry (FCM) was used to identify FSCT cells undergoing apoptosis induced by staurosporine (STS) (**Figure** **1**d), and ABs were isolated by differential centrifugation. The vesicles were washed away, and the ABs were centrifuged three times in PBS to eliminate protein residue (Figure 1a). Spherical ABs with a diameter of ≈1.5 µm were observed by scanning electron microscopy (SEM) (Figure 1b). The purity and size of the ABs were subsequently analyzed by FCM. Platelets of a size (1–4 µm) similar to the peritoneal thickness were used for comparison. The results of forward scatter/side scatter (FSC/SSC) analysis showed that ≈99.4% of the EVs that were obtained by differential centrifugation were ABs (Figure 1c). Western blotting (WB) revealed significantly higher levels of specific ABs markers, including histone H3 (H3), histone 2B (H2B), C1QC, C3B, CASP‐3 and cl‐CASP‐3, in the isolated ABs than in untreated FSCT cells (Figure 1e). ABs contain the distinct marker phosphatidylserine, which distinguishes them from other EVs.^[^ ^7a^ ^]^ FCM was used to identify unstained ABs as a negative control and determine the ABs’ positivity rate (Figure 1f). The results showed that 69.85% of EVs exhibited positive staining for phosphatidylserine, indicating that these EVs were ABs.

Various cell types are present in the flap, including vascular endothelial cells, macrophages, fibroblasts, and adipocytes. However, the promotion of flap survival relies mostly on increasing vascular proliferation and minimizing inflammation^[^ ^5^ ^]^; therefore, the pivotal roles of endothelial cells and macrophages were focused. Furthermore, to ensure the experimental validity of ABs in vitro and in vivo, bEnd.3 cells (endothelial cells) and Raw264.7 (macrophages) cells were respectively cocultured with DiI‐labelled ABs (20 µg mL^−1^) for 24 h. Subsequently, the presence of DiI‐positive cells were examined using microscopy and FCM, and the results indicated the internalization of ABs (Figure 1g,h). FCM revealed that ≈99.1% of endothelial cells and 96.9% of macrophages were positive for the uptake of DiI‐ABs. Immunofluorescence (IF) analysis showed that DiI‐ABs were visible in the cells. To assess the internalization of ABs in the flap in vivo, DiI‐labelled ABs were injected. Tissue samples on postoperative day 3 (POD3) were collected for frozen sectioning. Subsequently, separate CD31 and CD68 staining experiments were performed to examine the uptake of ABs by endothelial cells and macrophages within the tissue. Additionally, vesicle size and morphology were assessed (Figure 1i). The findings demonstrated the successful extraction of a high concentration of ABs from FSCT cells. Overall, endothelial cells and macrophages readily engulfed these larger vesicles in vitro and in vivo.

### 2.2 ABs Promoted Ischaemic Flap Survival In Vivo

**Figure 2:**
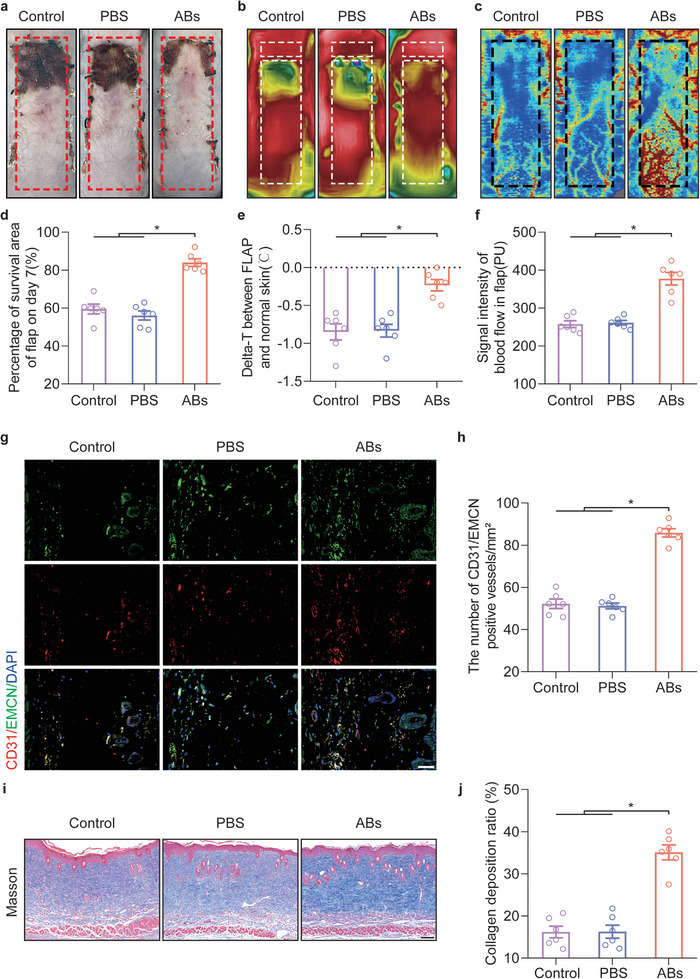
ABs promoted the survival of ischaemic flaps. a) Digital image of the flap survival area on POD7. b) Thermal images of the flap on POD7. c) Images of the subcutaneous blood flow network on POD7. d) Quantification of the percentage of the survival area in the three groups on POD7 (n = 6). e) Comparison of the delta‐T between FLAP skin and normal skin in the three groups on POD7. f) Quantification of the signal intensity indicating blood flow in ischaemic flaps in the three groups on POD7 (n = 6). g) IF staining of CD31 and EMCN in area II of the FLAP on POD7. Scale bar: 50 µm. h) Quantified CD31/EMCN‐positive blood vessel density in the three groups (n = 6). i) Masson staining was used to examine damaged collagen in the skin on POD7. Scale bar: 100 µm. j) Quantification of the Collagen deposition ratio in the three groups (n = 6). The error bars are the SEMs. Significance (*): *p value* < 0.05; ANOVA plus the LSD post hoc test (equal variances) or Dunnett's T3 method (unequal variances).

After the FLAP model was established on the backs of depilated C57BL/6 mice, ABs (1  mg mL^−1^, 100 µL) were subcutaneously injected into the FLAP with a microinjection needle, extending the axis at 8 points (Figure S2a, Supporting Information). The presence of avascular necrosis at the distal end of the flap was clearly observed on POD7 (**Figure** **2**a). Subcutaneous PBS injection had no impact on flap survival, while the survival area of the flap was significantly improved after subcutaneous injection of ABs compared with that in the Control group (Figure 2d). A real‐time infrared thermal imager was used to observe the skin flap seven days after surgery, and a temperature decrease was clearly observed in the necrotic area of the distal skin flap (Figure 2b). Considering that room temperature and anaesthetization of the mice could affect skin temperature, the relative temperature was chosen, which was the delta temperature (Delta‐T) between the FLAP and the neck (normal), as an index for observation. Additionally, the neck (normal) skin temperature on the back in each group was compared, and there were no significant differences (Figure S2b, Supporting Information). Compared with that in the Control group and the PBS group, the Delta‐T in the ABs group was markedly increased on POD7 (Figure 2e). In addition, laser doppler blood flow (LDBF) analysis revealed reconstruction of the subcutaneous vascular network on POD7 (Figure 2c). On POD7, the signals in the ABs group were markedly stronger than those in the Control and PBS groups, indicating skin blood flow (Figure 2f).

The FLAP was partitioned into three areas: area I was the proximal region with survival characteristics, area II was the area at the boundary of viable necrosis, and area III corresponded to the distal region where necrosis occurred. For further investigation, area II skin flaps on POD7 were used for tissue section staining. In the ABs group, a marked increase in the number of CD31/EMCN‐positive vessels was observed (Figure 2g,h). The WB results demonstrated that the expressions of the angiogenesis‐related proteins cadherin 5 and vascular endothelial growth factor (VEGF) were significantly increased in response to ABs (Figure S4a,b, Supporting Information). Furthermore, Masson staining showed that ABs injection promoted the remodeling of skin collagen (Figure 2i,j). In general, these findings suggested that subcutaneous injection of ABs promoted the repair of distal ischaemic flaps.

### 2.3 ABs Inhibited Oxidative Stress and Cell Death In Vivo

**Figure 3:**
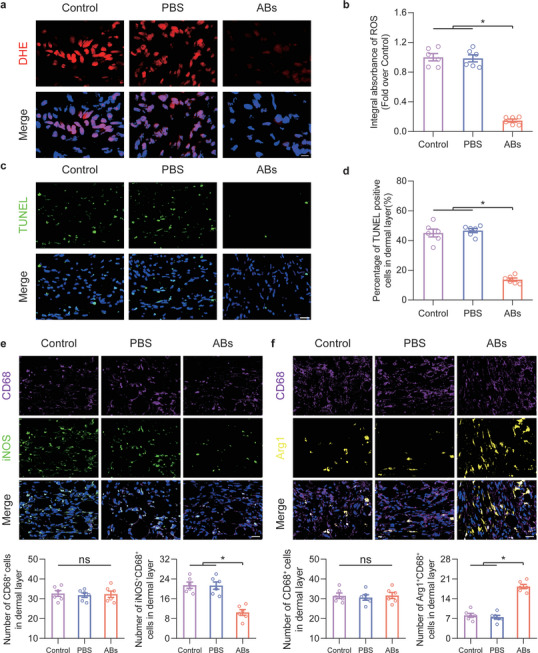
ABs inhibited oxidative stress and cell death and promoted the M1‐to‐M2 transition in ischaemic flaps. a) Frozen skin tissue sections in the three groups on POD7 were stained with DHE. Scale bar: 10 µm. b) Quantification of DHE staining in the three groups (n = 6). c) Dead cells in flap tissue sections on POD7 were examined by TUNEL staining. Scale bar: 20 µm. d) Quantification of the percentage of TUNEL‐positive cells in the dermal layer in the three groups (n = 6). e) CD68 and iNOS staining in area II of the flap in the three groups on POD7. Scale bar: 20 µm. The levels of infiltrated CD68^+^ macrophages and M1‐like (CD68^+^ and iNOS^+^) macrophages were quantified in the three groups (n = 6). f) CD68 and Arg1 staining in area II of the flap in the three groups on POD7. Scale bar: 20 µm. The levels of infiltrated CD68^+^ macrophages and M2‐like (CD68^+^ and Arg^+^) macrophages were quantified in the three groups (n = 6). The error bars are the SEMs. Significance (*): *p value* < 0.05; ns, not significant; ANOVA plus the LSD post hoc test (equal variances) or Dunnett's T3 test (unequal variances).

Oxidative stress levels and histiocyte death are typical pathological mechanisms of flap ischaemic injury and play major roles in the outcome of flap necrosis.^[^ ^4^, ^28^ ^]^ Therefore, it was investigated whether ABs could inhibit oxidative stress in ischaemic flaps and cell death. The level of oxidative stress in ischaemic flaps on POD 7 was examined using the ROS probe DHE (**Figure** **3**a). After the administration of ABs, ROS levels in the tissue were significantly reduced, and no obvious differences between the Control and PBS groups were observed (Figure 3b). Then, TUNEL staining was performed to examine the cells with broken DNA (dead cells),^[^ ^29^ ^]^ and TUNEL‐positive cells were observed (Figure 3c). The proportion of TUNEL‐positive cells was considerably reduced in the ABs group (Figure 3d).

Next, macrophage polarization in the flap was observed on POD7, and M1‐like (CD68^+^ and iNOS^+^) and M2‐like (CD68^+^ and Arg1^+^) macrophages were identified (Figure 3e,f). Compared with the Control and PBS groups, the ABs group exhibited a significantly reduced proportion of M1‐like macrophages but no change in the proportion of CD68^+^ macrophages. Moreover, the proportion of M2‐like macrophages was markedly increased in the ABs group. FCM analysis of the flap on POD7 showed that CD80‐positive M1 macrophages were significantly reduced in the ABs group (Figure S3a, Supporting Information), and CD206‐positive M2 macrophages were increased in the ABs group (Figure S3b, Supporting Information). Furthermore, cytokine chip testing was performed on the three groups. The PCA results showed that there was no obvious difference between the Control and PBS groups, and there was an intergroup difference between the ABs group and the Control and PBS groups (Figure S3c, Supporting Information). The cytokine profiles of the PBS group and the ABs group were compared, and a volcano plot was generated to show the relevant differentially expressed cytokines (Figure S3d, Supporting Information). The levels of macrophage‐related cytokines are shown on the heatmap (Figure S3e, Supporting Information). The expression of cytokines secreted by M1 macrophages was decreased, and the expression of cytokines secreted by M2 macrophages was increased. Accordingly, ABs suppressed oxidative stress and cell death levels and promoted M1‐to‐M2 polarization in macrophages in ischaemic flaps.

### 2.4 ABs Inhibited Ferroptosis In Vivo and In Vitro

**Figure 4:**
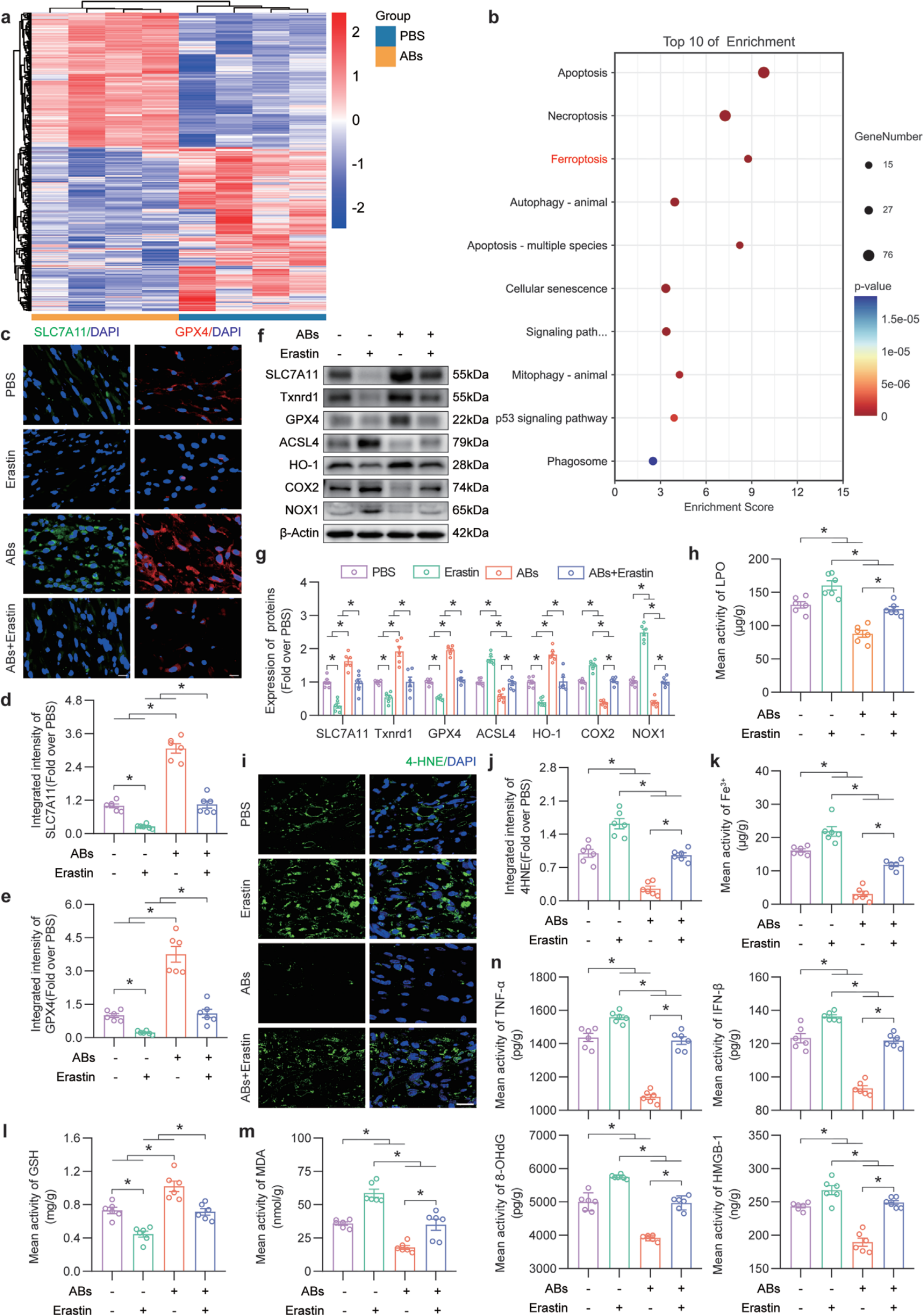
ABs inhibited ferroptosis in ischaemic flaps. a) Heatmap showing death, oxidative stress, and macrophage inflammation genes in the PBS group and ABs group (n = 4). b) Top 10 KEGG pathways enriched in GPX4‐GSH system‐related antioxidant genes. c) IF staining of SLC7A11 and GPX4 in FLAP area II in the three groups on POD7. Scale bars: 10 µm. d,e) Quantification of the integrated intensity of SLC7A11 (d) and GPX4 (e) staining in the dermal layer in the four groups (n = 6). f) Ferroptosis‐related protein levels in area II of the flap in the four groups on POD7. g) The quantified expression of ferroptosis‐related proteins in the skin of the four groups (n = 6) is shown on the right. h) LPO levels in area II of the flap on POD7 in the four groups (n = 6). i) IF staining of 4‐HNE in area II of FLAP on POD7 in the four groups. Scale bar: 20 µm. j) Quantified integrated intensity of 4‐HNE staining in the dermal layer in the four groups (n = 6). k) Quantification of the iron levels in area II of the FLAP in the four groups (n = 6). l,m) GSH (l) and MDA (m) levels in area II of the flap on POD7 in the four groups (n = 6). n) ELISA analysis of inflammatory factor (TNF‐*α*, IFN‐*β*) and DAMP (8‐OHdG, HMGB‐1) protein levels in area II of the FLAP in the four groups (n = 6). *β*‐Actin served as the loading control and for band density normalization. The error bars are the SEMs. Significance (*): *p value* < 0.05; ns, not significant; ANOVA plus the LSD post hoc test (equal variances) or Dunnett's T3 test (unequal variances).

In previous studies, high levels of oxidative stress were observed in ischaemic flaps on POD7.^[^ ^3^, ^4^ ^]^ Therefore, POD7 was considered to be of particular interest in flap research because it reflects flap survival. Transcriptome sequencing was performed on area II in the PBS and ABs groups on POD7. A statistical analysis was conducted to identify genes associated with cell death, oxidative stress, and macrophage inflammation, resulting in 653 genes (**Figure** **4**a). These genes underwent Kyoto Encyclopedia of Genes and Genomes (KEGG) enrichment analysis, with the top ten genes selected from the Cellular Processes section (Figure 4b). Then, WB and reagent test kit were performed to examine factors that are representative of various types of cell death: apoptosis (Bcl‐2 and Caspase‐3), pyroptosis (GSDMD‐N and Caspase‐1), necroptosis (MLKL and RIPK‐3), and ferroptosis (SLC7A11, GPX4, LPO and iron ions) (Figure S4c–h, Supporting Information). The results showed changes in the levels of indicators of apoptosis and ferroptosis. The level of GSDMD‐N, which is involved in pyroptosis, was changed, while the level of caspase‐1 remained unchanged. MLKL, a marker of necroptosis, exhibited altered expression, while RIPK‐3 expression remained unchanged (Figure S4c,d, Supporting Information). In response to ABs, the levels of indicators of various types of cell death completely or partially changed, indicating that ABs may have a stronger regulatory effect on apoptosis and ferroptosis than on pyroptosis or necroptosis. And the literature regarding ferroptosis in skin flaps is lacking, additional experiments were performed to examine ferroptosis in the flap. It is necessary to observe the level of ferroptosis in the flap, select the fresh flap at POD0 as a control, and detect lipid peroxidation (LPO) and iron accumulations in ischemic flaps on days 1, 3, 7, and 14 after surgery. As shown in Figure S5a (Supporting Information), as the postoperative time increases, compared with the POD0, the level of LPO in the ischaemic flap gradually increased and reached the highest level at POD7, which indicated the existence of LPO in the ischaemic flap. Then Figure S5b (Supporting Information) showed that the Iron ions level increased with time in ischaemic flaps and reached the highest level at POD7. To sum up, this suggested that ferroptosis was likely to exist in ischaemic flaps. Moreover, Fer‐1 (a ferroptosis inhibitor) was added as a control to compare the therapeutic effects of ABs. ABs and Fer‐1 both inhibited ferroptosis and promoted ischaemic flap survival, and the two exerted synergistic effects (Figure S5c–f, Supporting Information). Therefore, it was believed that inhibiting ferroptosis was a new treatment strategy for ischaemic flaps.

The WB results demonstrated that SLC7A11, a crucial protein involved in ferroptosis, was upregulated in response to exposure to ABs. Additionally, alterations in other ferroptosis‐related indicators were observed, indicating a decrease in the level of ferroptosis (Figure S4c–f, Supporting Information). As shown in Figure S6a,b (Supporting Information), after ABs treatment, the expressions of SLC7A11 in CD31‐positive endothelial cells and CD68‐positive macrophages in ischaemic skin flaps were increased. Moreover, the expression levels of GPX4 in endothelial cells and macrophages in ischaemic skin flaps were upregulated (Figure S6c,d, Supporting Information). These results indicated that ABs inhibited ferroptosis in ischaemic flaps and that ferroptosis was decreased in endothelial cells and macrophages. Ferroptosis occurs due to excessive accumulation of lipid peroxidation.^[^ ^30^ ^]^ The collapse of the Xc‐system plays a crucial role in increasing cells’ lipid peroxidation.^[^ ^14^ ^]^ And SLC7A11 is an important part of the Xc‐system.^[^ ^13^ ^]^ Therefore, it is necessary to determine whether ferroptosis was regulated by ABs through SLC7A11 modulation in flaps. Relevant literatures indicated that erastin inhibited SLC7A11 protein levels in endothelial cells^[^ ^31^ ^]^ and macrophage cells.^[^ ^32^ ^]^ Therefore, erastin was used as an SLC7A11 inhibitor in the flap model. The effect of ferroptosis on flaps was examined after injection of the erastin. IF analysis of SLC7A11 and GPX4 was performed (Figure 4c). As shown in Figure 4d,e, relative to that in the Control group, the expressions of SLC7A11 and GPX4 were suppressed in the Erastin group but increased in the ABs group, and the therapeutic effect of ABs was reversed by the combined application of ABs and erastin. In addition, WB revealed that changes in the levels of ferroptosis‐related proteins in the flap were reversed by the combined application of ABs and erastin (Figure 4f,g). Lipid peroxidation is a specific manifestation of ferroptosis.^[^ ^33^ ^]^ ELISA detecting of LPO and IF staining of 4‐hydroxynonenal (4‐HNE) were used to observe the level of lipid peroxidation in the flap. The level of LPO and intensity of 4‐HNE staining were markedly reduced in the ABs group, and this effect was reversed by erastin treatment (Figure 4h–j). Next, an iron assay was performed to examine iron levels in the tissue. Iron levels significantly decreased in the ABs group, and iron levels further increased in the ABs+Erastin group (Figure 4k). In addition, GSH and MDA assays revealed an increase in GSH expression in the ABs group, and this effect was reversed by erastin treatment (Figure 4l). On the other hand, MDA levels were decreased in response to ABs treatment, and in the ABs+Erastin group, the inhibitory effect on MDA levels was abolished (Figure 4m). The levels of inflammation‐related indicators (TNF‐*α* and IFN‐*β*) and DAMPs (8‐OHdG and HMGB‐1) were decreased in response to ABs treatment, and this effect was reversed by erastin (Figure 4n). Overall, these results indicated that ABs inhibited ferroptosis in ischaemic flaps by increasing SLC7A11.

Given the potential link between macrophage polarization and ferroptosis, experiments were performed to investigate this relationship. The macrophage scavenger clodronate liposomes from Vrije Universiteit Amsterdam (CL‐FVUA) was administered and a significant reduction in CD68‐positive macrophages were observed within the tissue (Figure S7a, Supporting Information). The results showed that the level of ferroptosis increased following macrophage clearance and was reduced by the administration of the Fer‐1, as evidenced by the analysis of tissue iron levels and IF analysis of SLC7A11 and 4‐HNE (Figure S7b–f, Supporting Information). These findings suggested a direct relationship between macrophages and ferroptosis in the skin flap. The results of IF showed that the ability of ABs to promote macrophage M1 to M2 polarization was reversed under the action of erastin in ischaemic flap (Figure S7g,h, Supporting Information). Moreover, a previous study revealed that macrophage M2 polarization effectively protected the survival of ischaemic flaps.^[^ ^34^ ^]^ Therefore, it was suggested that inhibiting the occurrence of ferroptosis by ABs in ischaemic flaps promoted flap survival via promoting M1 to M2 macrophages polarization.

Next, detections were conducted to determine whether the protective effect of ABs on ischemic flaps resulted from ferroptosis inhibition. In the presence of the ferroptosis inducer erastin, the ABs‐induced increase in the survival area of the flap was reversed (Figure S8a,b, Supporting Information). In addition, statistical analysis revealed that erastin reversed the suppressive effect of ABs on the delta‐T between the flap and normal skin (Figure S8d,e, Supporting Information). LDBF analysis showed that Erastin markedly suppressed the intensity of the signal, indicating blood flow in the flaps after ABs treatment (Figure S8c,f, Supporting Information). Furthermore, a dramatic decrease in the quantity of CD31/EMCN‐positive blood vessels was observed in the ABs+Erastin group (Figure S8g,i, Supporting Information). Masson staining showed that erastin reversed the remodeling of skin collagen induced by ABs (Figure S8h,j, Supporting Information).

To further verify how ABs affect the function of oxidatively damaged bEnd.3 cells, tert‐butyl hydroperoxide (TBHP) treatment was used to simulate the damaging oxidative environment of ischaemic skin flaps, and the appropriate TBHP concentration (110 µm) was determined by Cell Counting Kit‐8 (CCK‐8) experiments (Figure S9a, Supporting Information). Different concentrations of ABs were administered, and a CCK‐8 assay was performed to determine the optimal concentration for administration. The result indicated that the ABs concentration of 37.5 µg mL^−1^ was optimal, and chosen for in vitro experiments (Figure S9b, Supporting Information). CCK‐8 assay was also performed as the cytotoxicity experiment to detect the endothelial cells viability of NC, TBHP+PBS, TBHP+Erastin, TBHP+ABs and TBHP+ABs+Erastin groups, and results showed erastin reversed the ability of ABs to promote endothelial cell survival. (Figure S9c, Supporting Information). As shown in Figure S9d (Supporting Information), DiI was used to label the cell membrane, and the fluorescence intensity of SLC7A11(Xc‐) on the membrane was examined. The TBHP+ABs group exhibited restoration of the Xc‐ system on the membrane, whereas the effect of ABs was reversed by erastin treatment (Figure S9e, Supporting Information). The probe Ferro Orange was used to examine Fe^2+^ levels in the cells. IF analysis of Ferro Orange and 4‐HNE revealed the reversal of the therapeutic effect of ABs after erastin treatment (Figure S9f–i, Supporting Information). Taken together, these findings showed that ABs promoted ischaemic flap survival by inhibiting ferroptosis.

### 2.5 ABs Regulated the KEAP1‐Nrf2 Axis In Vivo

**Figure 5:**
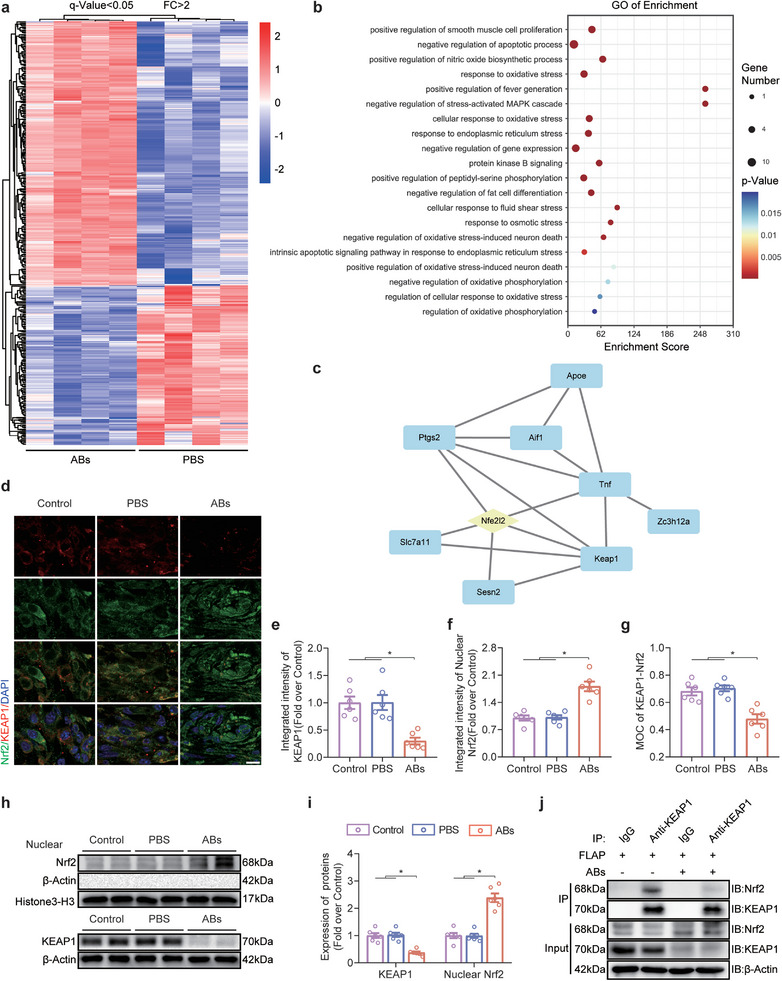
ABs inhibited KEAP1 and promoted Nrf2 nuclear translocation in ischaemic skin flaps. a) Heatmap showing differentially expressed Nrf2 target genes in the PBS group and ABs group (*p value* < 0.05; FC > 2). b) Bubble diagram of the top 20 genes related to the oxidative stress pathway enriched in differentially expressed Nrf2 target genes, as determined by GO pathway analysis. c) Network of interacting oxidative stress pathway‐related genes that were shown by GO enrichment analysis to be involved in the Nrf2‐KEAP1 axis. d) IF staining of KEAP1 and Nrf2 in FLAP area II on POD7 in the three groups. Scale bar: 10 µm. e–g) Quantified integrated intensity of KEAP1 (e) and nuclear Nrf2 (f) and the MOC for KEAP1‐Nrf2 (g) in the dermal layer in the three groups (n = 6). h) KEAP1 and nuclear Nrf2 protein levels in area II of the FLAP on POD7 in the three groups. i) Quantification of the protein expression of KEAP1 and nuclear Nrf2 in the four groups. *β*‐Actin and Histone 3‐H3 served as loading controls and for band density normalization, respectively (n = 6). j) Co‐IP analysis was used to examine the interaction between KEAP1 and Nrf2 in the skin in the three groups on POD7. The error bars are the SEMs. Significance (*): *p value* < 0.05; ANOVA plus the LSD post hoc test (equal variances) or Dunnett's T3 method (unequal variances).

Next we aimed to examine the upstream pathways by which ABs inhibit ferroptosis and promote flap survival. Several studies have confirmed that various types of EVs inhibit ferroptosis through Nrf2‐ or SLC7A11‐related mechanisms. For example, bone marrow stem cell‐derived EVs (exosomes and microvesicles) alleviate intervertebral disc degeneration in mice by suppressing ferroptosis through the circ_0072464/miR‐431/NRF2 axis.^[^ ^21a^ ^]^ Platelet‐derived EVs upregulate ITGB3 to increase the expression of SLC7A11 by increasing protein stability and activating the MAPK/ERK/ATF7/Nrf2 axis, which inhibits ferroptosis.^[^ ^21b^ ^]^ MSC‐Exos protect against ferroptosis by maintaining the functionality of SLC7A11.^[^ ^21c^ ^]^ Other types of EVs primarily modulate ferroptosis by activating Nrf2 or SLC7A11 expression; the difference lies in their upstream targets. It was postulated that Nrf2 was involved in the effect of ABs on the ischaemic flap. Nrf2, which is a putative antioxidative stress‐induced transcription factor, is not only responsible for the transcription of antioxidant‐related genes but also increases the expression of SLC7A11.^[^ ^35^ ^]^ Significant Nrf2‐targeted genes (q value < 0.05, FC > 2) identified based on transcriptome sequencing data were selected for Gene Ontology (GO) enrichment analysis (**Figure** **5**a). The GO results showed that the oxidative stress pathway was enriched, and some other pathways were selected (a total of 20), from which a bubble diagram was drawn (Figure 5b). Subsequently, a protein–protein interaction (PPI) map was generated to identify the interactions between the KEAP1, Nrf2, and SLC7A11 proteins in the presence of ABs (Figure 5c). The IF results demonstrated that in response to ABs, KEAP1 levels decreased in CD31‐ and CD68‐positive cells, while nuclear Nrf2 levels increased.

Then, IF analysis was performed to examine KEAP1‐Nrf2 in vivo (Figure 5d). The expression of KEAP1 in the flap was decreased in the ABs group (Figure 5e), and the expression of nuclear Nrf2 was increased (Figure 5f). Compared with that in the Control group and the PBS group, the Manders overlap coefficient (MOC) in the ABs group was significantly reduced, which suggested decreased binding between KEAP1 and Nrf2 in the ABs group (Figure 5g). The WB results showed that KEAP1 levels were decreased, while nuclear Nrf2 levels were increased in the ABs group (Figure 5h,i). Coimmunoprecipitation (Co‐IP) was then performed on the flaps in the PBS group and the ABs group, and the results showed that less Nrf2 was pulled down in the ABs group (Figure 5j). The IF colocalization analysis revealed that ABs significantly reduced the levels of KEAP1 and increased nuclear Nrf2 in endothelial cells and macrophages in the tissue (Figure S10, Supporting Information). Overall, ABs inhibited KEAP1 and activated the nuclear translocation of Nrf2 in vivo, and endothelial cells and macrophages exhibited changes in the KEAP1‐Nrf2 pathway.

### 2.6 ABs Inhibited Ferroptosis and Promoted Flap Survival via the KEAP1‐Nrf2 Axis

**Figure 6:**
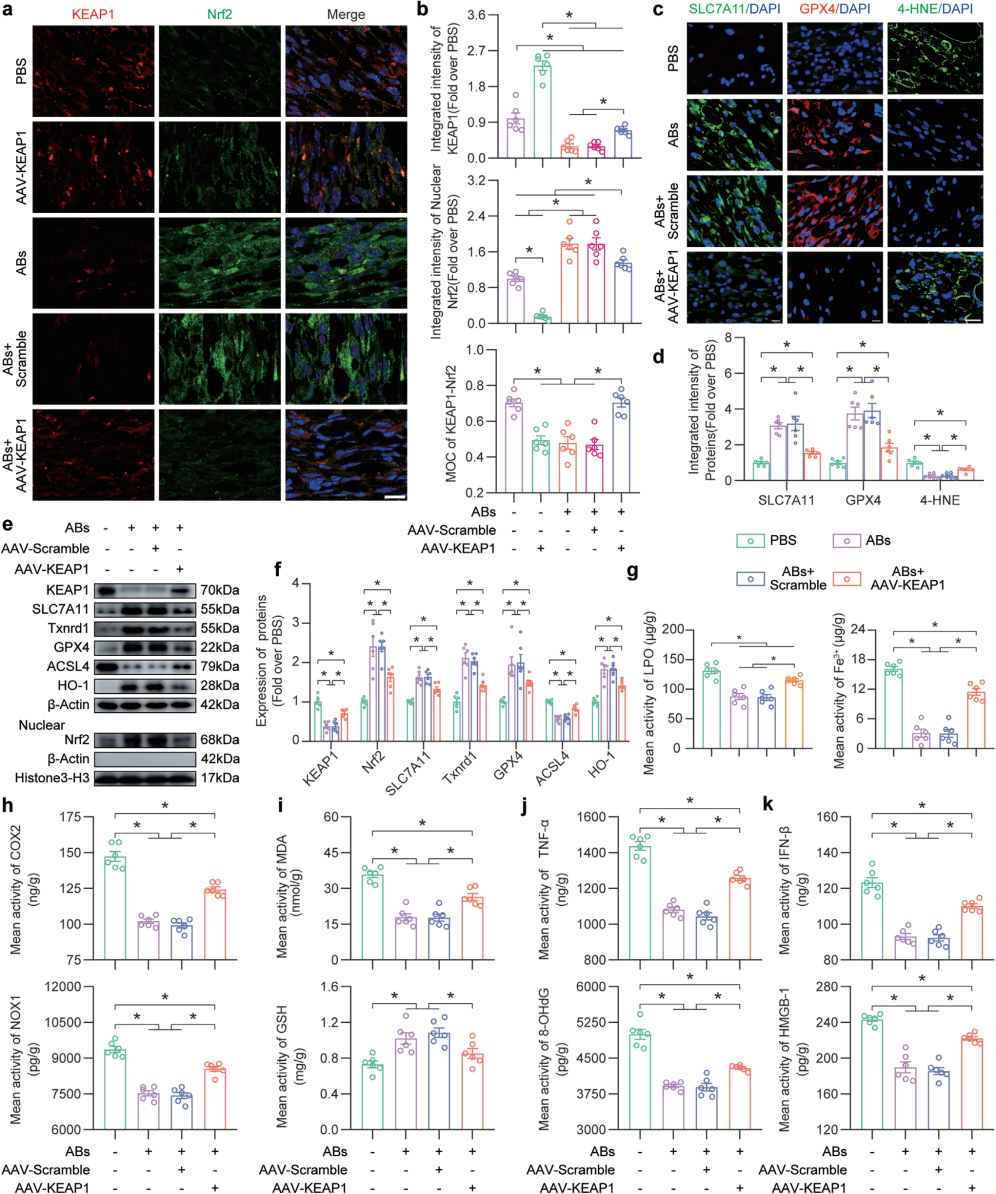
ABs inhibited ferroptosis in ischaemic flaps via the KEAP1‐Nrf2 axis. a) IF staining of KEAP1 and Nrf2 in FLAP area II on POD7 in the five groups. Scale bar: 10 µm. b) Quantified integrated intensity of KEAP1 (top) and nuclear Nrf2 (middle) and the MOC for KEAP1‐Nrf2 (bottom) in the dermal layer in the five groups (n = 6). c) IF staining of SLC7A11, GPX4 and 4‐HNE in FLAP area II on POD7 in the four groups. Scale bars: 10 µm (SLC7A11, GPX4) and 20 µm (4‐HNE). d) Quantification of the integrated intensity of SLC7A11, GPX4 and 4‐HNE in the dermal layer in the four groups (n = 6). e) KEAP1, nuclear Nrf2 and ferroptosis‐related protein levels in FLAP area II in the four groups on POD7. f) Comparison of the expression levels of KEAP1, nuclear Nrf2 and ferroptosis‐related proteins in the four groups. *β*‐Actin and Histone 3‐H3 served as loading controls for band density normalization (n = 6). g) Quantification of the LPO (left) and iron (right) levels in area II of the FLAP in the four groups (n = 6). h) ELISA analysis of area II of the FLAP to examine the COX2 (top) and NOX1 (bottom) levels in the four groups on POD7 (n = 6). i) MDA (top) and GSH (bottom) levels in area II of the flap in the four groups on POD7 (n = 6). j) ELISA analysis of TNF‐*α* (top) and 8‐OHdG (bottom) levels in area II of the flap in the four groups on POD7 (n = 6). k) ELISA of IFN‐*β* (top) and HMGB‐1 (bottom) levels in area II of the flap in the four groups on POD7 (n = 6). The error bars are the SEMs. Significance (*): *p value* < 0.05; ANOVA plus the LSD post hoc test (equal variances) or Dunnett's T3 method (unequal variances).

To confirm how KEAP1 inhibition regulates ABs‐induced ferroptosis, AAV‐KEAP1 was used to overexpress KEAP1 (**Figure** **6**a). The IF results showed that the ABs+AAV‐KEAP1 group expressed more KEAP1 than the ABs group or the ABs + Scramble group, and the ability of the ABs to inhibit KEAP1 was reversed by AAV‐KEAP1 (Figure 6a,b, top). In addition, there was increased nuclear Nrf2 expression in the ABs group and the ABs + Scramble group, but this change was reversed by AAV‐KEAP1 (Figure 6b, middle). Compared with those in the PBS group and the ABs + AAV‐KEAP1 group, the MOC values of the AAV‐KEAP1 group, the ABs group and the ABs + AAV‐KEAP1 group were lower (Figure 6b, bottom). The WB results showed that compared with those in the ABs and ABs + Scramble groups, tissue KEAP1 levels were increased after the application of AAV‐KEAP1, while nuclear Nrf2 levels were decreased (Figure 6e–f).

Further in vivo research was needed to examine the effect of the KEAP1‐Nrf2 axis on ferroptosis. The IF results were shown in Figure 6c,d and suggested that SLC7A11 and GPX4 levels in the flaps were decreased in the ABs + AAV‐KEAP1 group, and the level of 4‐HNE was increased in the ABs + AAV‐KEAP1 group. WB analysis of ferroptosis‐related proteins revealed that the inhibitory effect of ABs on ferroptosis was reversed by AAV‐KEAP1 (Figure 6e,f). Iron and LPO accumulations were higher in the skin flaps of AAV‐KEAP1‐treated mice than in those in the ABs group or the ABs + Scramble group (Figure 6g). The ELISA results revealed a change in the amounts of COX2 and NOX1 in the tissues in the ABs + AAV‐KEAP1 group. In addition, the ability of ABs to increase GSH and decrease MDA levels in ischaemic skin flaps was inhibited by KEAP1 overexpression (Figure 6i). The ABs‐AAV‐KEAP1 group exhibited a reversal of changes in tissue inflammation and the levels of DAMPs, which were used as indicators (Figure 6j,k). Overall, ABs inhibited ferroptosis through the KEAP1‐Nrf2 axis in ischaemic skin flaps.

Next, we examined whether the protective effects of ABs on ischaemic skin flaps were achieved through the KEAP1‐Nrf2 axis. The DHE staining results showed that AAV‐KEAP1 reversed the inhibitory effect of ABs on ROS (Figure S11a,b, Supporting Information). TUNEL staining revealed more apoptotic cells in the ABs+AAV‐KEAP1 group than in the ABs or ABs + Scramble groups (Figure S11c,d, Supporting Information). The results of macrophage IF staining showed that ABs promoted macrophage polarization from the M1 to the M2 phenotype by inhibiting KEAP1 (Figure S11e,f, Supporting Information). As shown in Figure S10a,b (Supporting Information), the ABs‐induced increase in flap survival was reversed by the overexpression of KEAP1. Statistical analysis revealed that AAV‐KEAP1 reversed the inhibitory effect of ABs on the delta‐T between FLAP skin and normal skin (Figure S12d,e, Supporting Information). LDBF analysis also showed that KEAP1 overexpression markedly suppressed the intensity of the signal, indicating blood flow in the flaps after ABs treatment (Figure S12c,f, Supporting Information). Furthermore, a marked decrease in the number of CD31/EMCN‐positive vessels was observed in the ABs+AAV‐KEAP1 group (Figure S12g,i, Supporting Information). Masson staining showed that the increase in KEAP1 reversed the remodeling of skin collagen induced by ABs (Figure S12h,j, Supporting Information). Taken together, these results suggested that ABs promoted ischaemic flap survival through the KEAP1‐Nrf2 axis.

### 2.7 ABs Targeted KEAP1 for Inhibition by Delivering miR‐339‐5p In Vivo

**Figure 7:**
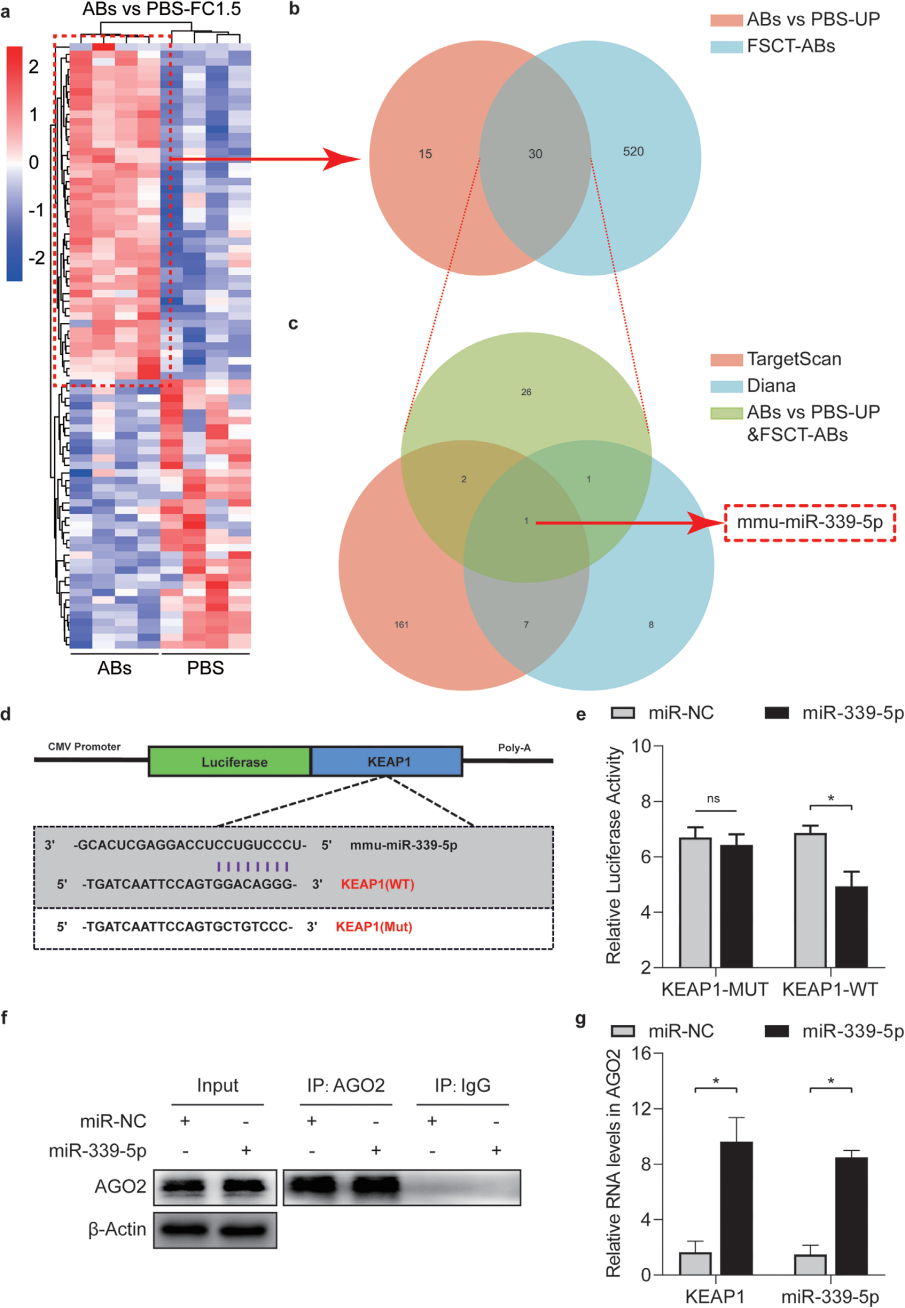
ABs upregulated miR‐339‐5p, which targeted KEAP1, in ischaemic skin flaps. a) Heatmap showing differentially expressed miRs in the flaps of mice treated with PBS and ABs on POD 7. b) Venn diagram showing that miRs with increased expression in the heatmap (a) overlapped with FSCT‐ABs. c) Venn diagram showing the overlap between KEAP1‐targeting miRs identified by TargetScan and Diana and the upregulated miRs in (b). d,e) Luciferase reporter assay to compare the relative luciferase activity in 293T cells after two days of treatment with *KEAP1*‐WT, *KEAP1*‐MUT, *miR‐NC* or a *mmu‐miR‐339‐5p* mimic (n = 5). f) IP of the RISC in miR‐NC‐ or miR‐339‐5p‐overexpressing bEnd.3 cells using the pan‐AGO2 antibody. IgG and *β*‐actin served as negative controls and internal controls, respectively. g) Relative incorporation of KEAP1 and mmu‐miR‐339‐5p into the RISC in miR‐NC‐ or miR‐339‐5p‐overexpressing bEnd.3 cells (n = 5). The error bars are the SEMs. Significance (*): *p value* < 0.05; ns, not significant; two‐tailed, unpaired t test.

Among all EV subtypes, ABs contain the highest concentration of miRs. miRs play a prominent role in the ability of ABs to promote flap survival.^[^ ^27^ ^]^ Furthermore, miRs were identified that targeted KEAP1 through the uptake of ABs in vivo. POD7 was selected for comparison. POD7 was considered to be of particular interest in flap research because it reflects flap survival.^[^ ^3^, ^4^, ^28^ ^]^ Therefore, miR‐sequencing of FSCT‐ABs and flap tissue in the PBS and ABs groups (n = 4) on POD7 was performed. We selected miRs that were upregulated in the tissue and were consistent with the miRs in FSCT‐ABs (**Figure** **7**a,b). A total of 30 identical miRs were selected. Then, the TargetScan and Diana databases were searched for miRs that were predicted to target KEAP1. Venn diagrams show that after these three sets of miRs were compared, mmu‐miR‐339‐5p was predicted to target KEAP1 in vivo (Figure 7c).

To further verify the targeted repression of KEAP1 by mmu‐miR‐339‐5p, WT and MUT 3′‐UTR sequences of *KEAP1* were constructed (Figure 7d), and subsequent experiments were performed. Then, a luciferase reporter gene was examined in transgenic 293T cells. The relative luciferase activity was markedly reduced when mmu‐miR‐339‐5p was upregulated and when the cells were cotransfected with the WT *KEAP1* luciferase construct instead of the MUT *KEAP1* luciferase construct (Figure 7e). The mRNA level of *KEAP1* in the RNA‐induced silencing complex (RISC) after mmu‐miR‐339‐5p overexpression was examined by qPCR analysis (Figure 7f). Cells overexpressing mmu‐miR‐339‐5p exhibited high *KEAP1* levels, and *KEAP1* mRNA was integrated into the RISC in these cells (Figure 7g). Overall, these findings indicated that *KEAP1* was a target of mmu‐miR‐339‐5p.

### 2.8 ABs Inhibited Ferroptosis and Promoted Ischaemic Flap Survival via miR‐339‐5p In Vivo

To further examine whether ABs target KEAP1 to promote ischaemic flap survival in vivo through the delivery of miR‐339‐5p, miR‐339‐5p was knocked down or overexpressed in bEnd.3 cells via a lentiviral vector (LV). qPCR verified that the amount of miR‐339‐5p changed when miR‐339‐5p was inhibited (ABs‐miR‐339‐5p in) or overexpressed (ABs‐miR‐339‐5p oe) in ABs (Figure S13a, Supporting Information). Figure S1 (Supporting Information) shows the characterization of ABs‐miR‐339‐5p in and ABs‐miR‐339‐5p oe. The levels of surface markers were different from those of ABs themselves. To control for the effect of alternative pathways that mediate the changes induced by the LV, ABs‐LV‐miR‐339‐5p, and ABs‐siRNA‐miR‐339‐5p (in which the siRNA was used to target miR‐339‐5p) were compared. The qPCR results showed that miR‐339‐5p levels were reduced in both groups (Figure S13b, Supporting Information). Furthermore, when exposed to ABs‐LV‐miR‐339‐5p or ABs‐siRNA‐miR‐339‐5p, the surface area of the skin flap decreased moderately (Figure S13c,d, Supporting Information). Additionally, ABs‐LV‐miR‐339‐5p exposure inhibited KEAP1 by comparable levels and increased the level of nuclear Nrf2 (Figure S13e,f, Supporting Information). The parent cell line induced by LV produced more stable cells compared to those produced with siRNA, without altering the efficacy of the miR‐339‐5p/KEAP1/Nrf2 pathway. Thus, ABs‐miR‐339‐5p produced by LV‐transfected cells and ABs‐miR‐339‐5p oe were used for subsequent investigations.

**Figure 8:**
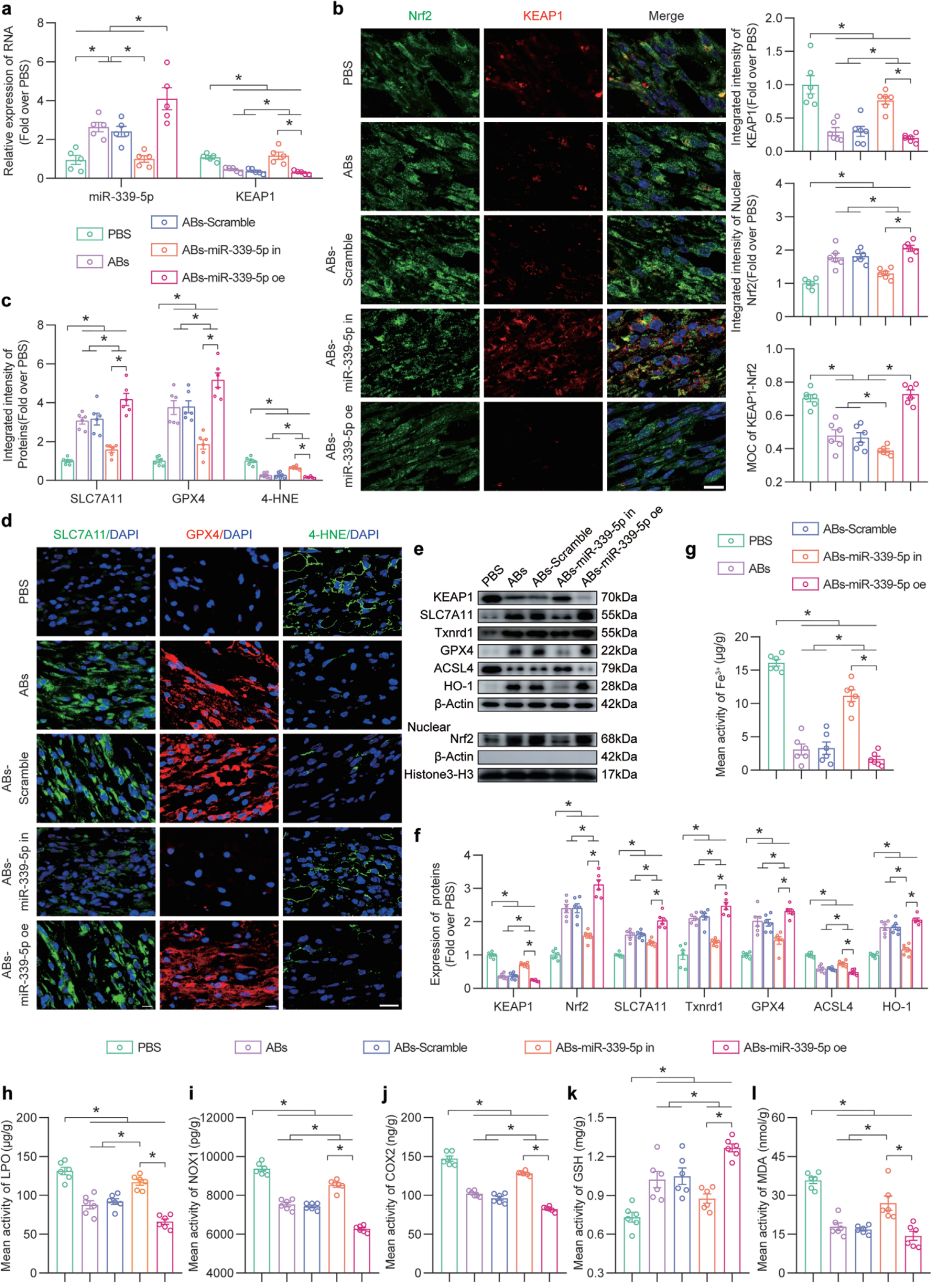
ABs inhibited ferroptosis in ischaemic skin flaps via the miR‐339‐5p/KEAP1 axis. a) Comparison of the relative expression levels of KEAP1 and miR‐339‐5p in the skin in the five groups on POD7 (n = 5). b) IF staining of KEAP1 and Nrf2 in FLAP area II in the five groups on POD7. Scale bar: 10 µm. The integrated intensity of KEAP1 (top) and nuclear Nrf2 (mid) and the MOC for KEAP1‐Nrf2 (bottom) in the dermal layer of the five groups were quantified (n = 6). c) Quantified integrated intensity of SLC7A11, GPX4 and 4‐HNE in the dermal layer in the five groups (n = 6). d) IF staining of SLC7A11, GPX4 and 4‐HNE in FLAP area II in the five groups on POD7. Scale bars: 10 µm and 20 µm (4‐HNE). e) KEAP1, nuclear Nrf2 and ferroptosis‐related protein levels in FLAP area II in the five groups on POD7. f) Comparison of the expression levels of KEAP1, nuclear Nrf2 and ferroptosis‐related proteins in the skin in the five groups. *β*‐Actin and Histone 3‐H3 served as loading controls and for band density normalization, respectively (n = 6). g) Quantification of the iron levels in area II of the FLAP in the five groups (n = 6). h‐j) ELISA analysis of LPO (h), NOX1 (i) and COX2 (j) levels in area II of the flap in the five groups on POD7 (n = 6). k,l) GSH (k) and MDA (l) levels in area II of the flap in the five groups on POD7 (n = 6). The error bars are the SEMs. Significance (*): *p value* < 0.05; ANOVA plus the LSD post hoc test (equal variances) or Dunnett's T3 method (unequal variances).

Flap tissue was assessed by qPCR, and the ABs and ABs‐LV‐scramble (ABs‐Scramble) groups exhibited high miR‐339‐5p expression and decreased *KEAP1* expression. In the ABs‐miR‐339‐5p in group, the expression of miR‐339‐5p was inhibited, and *KEAP1* was increased. Additionally, the ABs‐miR‐339‐5p oe group expressed more miR‐339‐5p and less *KEAP1* (**Figure** **8**a). IF analysis revealed that compared with the ABs, ABs‐scramble and ABs‐miR‐339‐5p oe groups, the ABs‐miR‐339‐5p in group expressed more KEAP1 and significantly less nuclear Nrf2, and the MOC value for KEAP1‐Nrf2 increased (Figure 8b). Similarly, the WB results showed similar changes in KEAP1 and nuclear Nrf2 levels (Figure 8e,f). ELISA was used to examine COX2 and NOX1 expression, and the results were consistent with the WB results (Figure 8i,j). The IF results are shown in Figure 8c,d and revealed that miR‐339‐5p‐inhibited ABs did not increase the levels of SLC7A11 or GPX4 in the flaps and maintained the increase in 4‐HNE expression. In addition, compared with those in the ABs, ABs‐scramble and ABs‐miR‐339‐5p oe groups, the changes in the expression of ferroptosis‐related proteins in the ABs‐miR‐339‐5p in group were reversed, as shown by WB (Figure 8e,f). Moreover, after miR‐339‐5p was knocked down, the flaps exhibited more iron and LPO deposition, and less iron and LPO deposition was observed after miR‐339‐5p was overexpressed (Figure 8g,h). Furthermore, the ability of ABs to increase GSH levels and decrease MDA levels in ischaemic flaps was reversed after the knockdown of miR‐339‐5p, and these changes were more pronounced after miR‐339‐5p overexpression (Figure 8i,j). ABs‐miR‐339‐5p in reversed the changes in tissue inflammation and the expression of DAMPs, which were used as indicators (Figure S14, Supporting Information). Overall, these findings showed that ABs inhibited ferroptosis in ischaemic skin flaps via the miR‐339‐5p/KEAP1/Nrf2 pathway.

Next, we aimed to determine whether the protective effects of ABs on oxidative stress and cell death were mediated by miR‐339‐5p. DHE staining showed that more ROS accumulated in the ABs‐miR‐339‐5p in group than in the ABs or ABs‐Scramble group (Figure S15a,b, Supporting Information). TUNEL staining suggested that miR‐339‐5p knockdown reversed the inhibitory effect of ABs on cell death (Figure S15c,d, Supporting Information). Additionally, the proportion of M1‐like macrophages was significantly increased in the ABs‐miR‐339‐5p in group compared to the ABs and ABs‐Scramble groups, while the proportion of CD68^+^ macrophages was unchanged (Figure S13e, Supporting Information). Moreover, the number of M2‐like macrophages in the ABs‐miR‐339‐5p in group was significantly reduced (Figure S15f, Supporting Information). ABs‐miR‐339‐5p oe group exhibited robust inhibition of oxidative stress and cell death and increased M2 macrophage polarization. Overall, these findings suggested that ABs reduced ROS levels, inhibited cell death, and promoted M2 macrophage polarization in ischaemic skin flaps by delivering miR‐339‐5p.

It was further examined whether ABs promoted the survival of ischaemic flaps by delivering miR‐339‐5p. As shown in Figure S16a,b (Supporting Information), the flap survival area was reduced after miR‐339‐5p was inhibited in ABs. Statistical analysis revealed that the miR‐339‐5p inhibitor reversed the effect of ABs on the Delta‐T between FLAP skin and normal skin (Figure S16c,d, Supporting Information). LDBF analysis also showed that after ABs treatment, miR‐339‐5p knockdown suppressed the signal intensity, indicating blood flow in the flaps (Figure S16e,f, Supporting Information). Furthermore, fewer CD31/EMCN‐positive vessels were observed in the ABs‐miR‐339‐5p in group (Figure S14g,h, Supporting Information). Masson staining suggested that the knockdown of miR‐339‐5p reversed the remodeling of skin collagen induced by ABs (Figure S16i,j, Supporting Information). Although there was no significant increase in vascular density after the overexpression of miR‐339‐5p compared to that in the ABs group, miR‐339‐5p overexpression promoted further survival and collagen remodeling in the flap. Taken together, these results suggested that ABs promoted ischaemic flap survival by delivering miR‐339‐5p.

### 2.9 ABs Inhibited Ferroptosis and Promoted Endothelial Cell Survival via miR‐339‐5p/KEAP1 In Vitro

**Figure 9:**
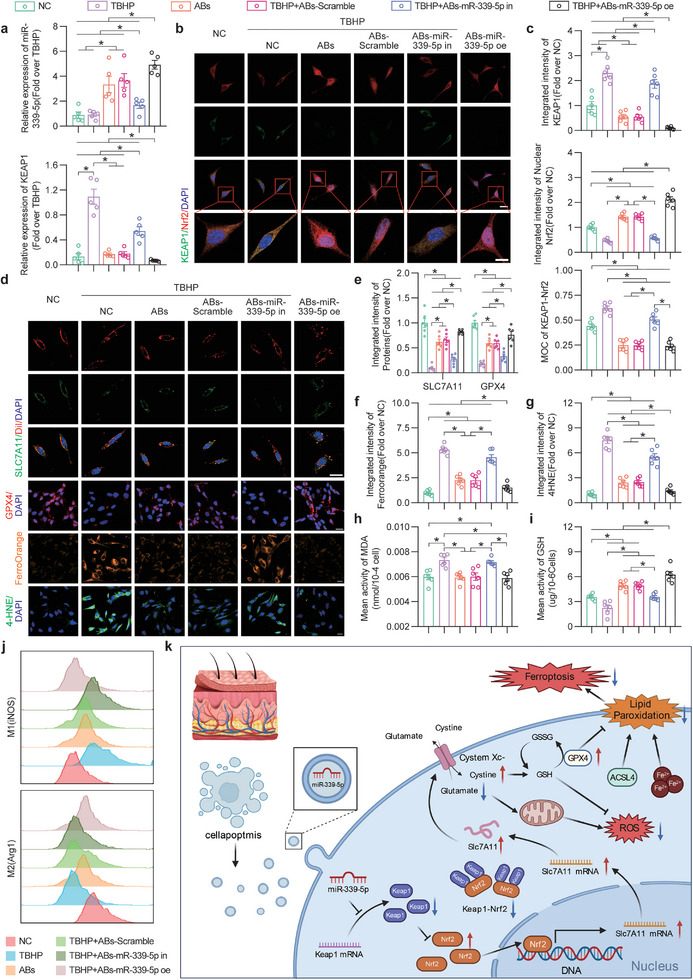
miR‐339‐5p knockdown reversed the ability of ABs to inhibit KEAP1 expression and ferroptosis in bEnd.3 cells. a) Comparison of the relative expression levels of KEAP1 (bottom) and miR‐339‐5p (top) in bEnd.3 cells in the six groups after treatment for 24 h (n = 6). b) IF staining of KEAP1 and Nrf2 in bEnd.3 cells in the six groups after treatment for 24 h. Scale bars: 20 and 10 µm. c) Quantified integrated intensity of KEAP1 (top) and nuclear Nrf2 (mid) and the MOC for KEAP1‐Nrf2 (bottom) in bEnd.3 cells in the six groups after treatment for 24 h (n = 6). d) IF staining of SLC7A11/DiI and GPX4 and Ferro Orange and 4‐HNE staining in bEnd.3 cells in the six groups after treatment for 24 h. Scale bars: 20 µm. e) Quantification of the integrated intensity of SLC7A11/DiI and GPX4 in bEnd.3 cells in the six groups after treatment for 24 h (n = 6). f,g) Quantified integrated intensity of Ferro Orange (f) and 4‐HNE (g) staining in the six groups after treatment for 24 h (n = 6). h,i) The MDA (h) and GSH (i) levels in bEnd.3 cells in the six groups after treatment for 24 h (n = 6). j) FCM of M1 (iNOS, top) and M2 (Arg1, bottom) Raw264.7 cells in the six groups after treatment for 24 h. k) Schematic showing the molecular mechanism of FSCT cell‐derived ABs, emphasizing the functions of miR‐339‐5p, the KEAP1/Nrf2 axis, and ferroptosis in the treatment of ischaemic flap necrosis. The error bars are the SEMs. Significance (*): *p value* < 0.05; ANOVA plus the LSD post hoc test (equal variances) or Dunnett's T3 method (unequal variances).

qPCR revealed high miR‐339‐5p expression in the TBHP+ABs, TBHP+ABs‐Scramble and TBHP+ABs‐miR‐339‐5p oe groups and decreased intracellular miR‐339‐5p levels after the use of ABs to knock down miR‐339‐5p (**Figure** **9**a, top). When miR‐339‐5p was inhibited, intracellular *KEAP1* levels increased (Figure 9a, bottom). IF staining revealed that after ABs were used to knock down miR‐339‐5p, KEAP1 expression increased, nuclear Nrf2 expression decreased, and the MOC value for KEAP1‐Nrf2 increased (Figure 9b,c).

As shown in Figure 9d, the Xc‐ system on the membrane was restored in the TBHP+ABs and TBHP+ABs‐Scramble groups, and this effect of ABs was reversed after miR‐339‐5p knockdown (Figure 9e). IF analysis of GPX4 revealed that the therapeutic effect of ABs was reversed after miR‐339‐5p inhibition (Figure 9d,e). Furthermore, JC‐1 staining was used to observe the mitochondrial membrane potential levels in each group (Figure S17c, Supporting Information). In response to TBHP injury, the ratio of mitochondrial monomers to aggregates decreased, suggesting that the mitochondrial membrane potential decreased; the membrane potential was partially restored after the administration of ABs but did not recover after the administration of miR‐339‐5p‐knockdown ABs (Figure S17d, Supporting Information). Iron accumulation was inhibited in the ABs and ABs‐Scramble groups, but iron accumulation was not inhibited in the ABs‐miR‐339‐5p in group (Figure 9d,f). The results of 4‐HNE staining showed that the ability of ABs to inhibit lipid peroxidation was reversed after the knockdown of miR‐339‐5p (Figure 9d,g). Furthermore, the therapeutic effect of ABs was further enhanced by the overexpressing of miR‐339‐5p. These results suggested that ABs inhibited ferroptosis in oxidatively damaged endothelial cells by delivering miR‐339‐5p.

The degree of oxidative stress‐induced damage repair was determined by DHE staining and analysis of intracellular GSH and MDA levels. As shown in Figure 9h,i, the ability of ABs to increase GSH and decrease MDA levels in vitro was reversed by miR‐339‐5p knockdown. The DHE staining results showed that ABs strongly inhibited the accumulation of ROS in vitro, and ROS levels increased after miR‐339‐5p knockdown (Figure S17a,b, Supporting Information). Furthermore, bEnd.3 cell activity was detected by in vitro angiogenesis (tube formation), cell migration, and scratch assays. As shown in Figure S17e,f (Supporting Information), angiogenesis was significantly increased in the TBHP+ABs and TBHP+ABs‐Scramble groups; however, the ABs‐miR‐339‐5p in group did not exhibit these effects. The cell migration assay showed that ABs effectively promoted cell migration, and the number of migrating cells was significantly reduced after the knockdown of miR‐339‐5p (Figure S17g,h, Supporting Information). The scratch assay showed that cell migration and repair capacity were partially restored after the administration of ABs and ABs‐Scramble, while treatment with miR‐339‐5p‐knockdown ABs did not achieve this effect (Figure S17i,j, Supporting Information). After the overexpression of miR‐339‐5p, MDA levels and the scratch assay showed no significant changes compared to that in the ABs group; however, the remaining indicators were superior to those in the ABs group. The polarization of Raw264.7 cells was assessed using FCM. ABs facilitated the polarization of macrophages from the M1 to the M2 phenotype. The ABs‐miR‐339‐5p oe group exhibited enhanced polarization; however, this effect was not observed for the ABs‐miR‐339‐5p in group (Figure 9j). Taken together, these results indicated that ABs inhibited KEAP1 expression and ferroptosis, maintained cell viability and facilitated the polarization of macrophages from the M1 to the M2 phenotype by delivering miR‐339‐5p.

## 3 Discussion

Flap transplantation is the best treatment method to repair large wounds.^[^ ^36^ ^]^ However, none of the available solutions for preventing ischaemic necrosis at the distal end of the flap are ideal. Oxidative stress damage, inflammation, and cell death due to flap ischaemia eventually led to necrosis of the distal end of the flap if not managed properly.^[^ ^3^ ^]^ We investigated whether artificially increasing the number of ABs derived from tissue cells adjacent to ischaemic flaps could promote flap survival. Our research revealed that with the administration of artificial FSCT cell‐derived ABs during surgery effectively promoted the survival of ischaemic flaps. We successfully isolated ABs from FSCT cells and confirmed their identity using specific (phosphatidylserine, CASP‐3 and cl‐CASP‐3) and nonspecific (size, aggregation and surface marker protein levels) markers. Various cell types are present in the flap, including vascular endothelial cells, macrophages, fibroblasts, and adipocytes. However, promoting flap survival relied mostly on increasing vascular proliferation and minimizing inflammation^[^ ^5^ ^]^; therefore, we focused on the pivotal roles of endothelial cells and macrophages. We successfully traced the localization of DiI‐labelled ABs in endothelial cells and macrophages in vitro and in vivo. These results served as the foundation for subsequent experiments.

First, we examined angiogenesis and oxidative stress, which are key components of flap ischaemic injury.^[^ ^3^, ^4^ ^]^ The accumulation of ROS is caused by an imbalance between increased ROS production and antioxidant capacity and affects the function of cells in the tissue.^[^ ^37^ ^]^ Previous studies have shown that ischaemic flap survival is promoted by reducing the accumulation of ROS.^[^ ^3a^ ^]^ Therefore, it was investigated whether ABs, which are a type of EV, inhibited the level of ROS in ischaemic flaps. ABs produced by the apoptosis of surrounding cells can be engulfed by cells and promote their proliferation.^[^ ^9^ ^]^ Therefore, we examined whether increasing these ABs could promote the survival of ischaemic flaps using FSCT cell‐derived ABs. Fluorescent probes were used to examine ROS, and the results showed that ABs robustly decreased ROS levels in ischaemic flaps. After the application of ABs, the subcutaneous vascular network increased in abundance, and angiogenesis indicators also increased. The level of cell death in ischaemic flap tissue also reflects flap survival. TUNEL staining revealed that, the number of dead cells in the flap was reduced in the presence of ABs. Inflammation, which is mainly caused by macrophages, is activated after ischaemic tissue injury.^[^ ^38^ ^]^ M1 macrophages cause inflammation, and M2 macrophages inhibit inflammation and promote repair.^[^ ^39^ ^]^ The results of this study demonstrated that ABs decreased the proportion of M1 macrophages and promoted the generation of M2 macrophages. These results suggested that ABs effectively inhibited oxidative stress, reduced cell death and promoted the transition of macrophages from the M1 phenotype to the M2 phenotype. We performed ischemic flap transcriptome sequencing on POD7 and screened for analysis of differentially expressed genes related to oxidative stress, cell death, and macrophages. Apoptosis, necroptosis and ferroptosis were positively expressed in KEGG enrichment analysis. WB analysis of various types of PCD suggested that ferroptosis played a key role in this process. So, we further investigated the regulation of ferroptosis by ABs in ischaemic flaps.

Ferroptosis is one of the most recently described forms of PCD,^[^ ^35^ ^]^ but its role in ischaemic flaps remains unclear. If necrosis occurs at the remote end of the skin flap, patients will need to undergo secondary surgery, which bears risk and results in great losses in terms of patient mental well‐being, physiology and financial costs. The occurrence of ferroptosis is closely related to the accumulation of ROS.^[^ ^12a^ ^]^ In this study, we first observed that ferroptosis was inhibited in endothelial cells and macrophages in ischaemic flaps treated with ABs. The administration of ABs significantly reduced the expression of ferroptosis‐related proteins, iron deposition and membrane lipid peroxidation. The ferroptosis activator erastin reversed these effects, highlighting the effectiveness of ABs in inhibiting ferroptotic cell death due to ischaemia in the flap. Additionally, the macrophage scavenger erastin and the ferroptosis inhibitor Fer‐1 were used to elucidate the link between ferroptosis and macrophage polarization. Our findings revealed that ABs suppressed ferroptosis and facilitated macrophage polarization from the M1 phenotype to the M2 phenotype. Furthermore, the decreases in inflammatory factors (TNF‐*α* and IFN‐*β*) and DAMPs (8‐OHdG and HMGB‐1) caused by ABs support this hypothesis. However, the specific mechanisms by which ABs exert protective effects on ischaemic flaps and inhibit ferroptosis require further study.

To elucidate the mechanism underlying the therapeutic effect of ABs on ischaemic flaps, upstream antioxidant, anti‐inflammatory and anti‐ferroptotic mechanisms were investigated. The study has shown that EVs attenuate ferroptosis by modulating Nrf2, thereby promoting the upregulation of SLC7A11, which is an antioxidant gene.^[^ ^21^ ^]^ We postulated that Nrf2 as involved in the ischaemic flap response to ABs and analyzed Nrf2‐associated target genes and pathways. Subsequently, we generated a PPI map to identify interactions between the KEAP1, Nrf2, and SLC7A11 proteins in the presence of ABs. KEAP1 was inhibited, and nuclear Nrf2 expression was increased in endothelial cells and macrophages after the administration of ABs to ischaemic flaps. We also assessed the expression levels of KEAP1 and Nrf2 and the binding between these proteins in ischaemic flaps. The results showed that ABs inhibited KEAP1 and increased the level of nuclear Nrf2. After the use of AAV‐KEAP1 to increase KEAP1 expression, ABs inhibited oxidative stress, inhibited ferroptosis, and activated the polarization of M1 macrophages to the M2 phenotype by inhibiting KEAP1. Overall, these results suggested that ABs protected ischaemic flaps by inhibiting KEAP1.

Considering that ABs exert therapeutic effects, a clearer demonstration of how ABs regulate KEAP1 activity and practical and effective evidence for further clinical applications in the future are needed. Interestingly, as a subtype of EV, ABs are produced only by apoptotic cells and retain a substantial number of characteristics from the parent cell, such as nucleic acid content, which also explains why ABs carry the most miRs among all EV subtypes.^[^ ^6^, ^7^, ^24^ ^]^ MiRs, which are a class of short, noncoding RNAs that mediate posttranscriptional regulation, precisely target and effectively silence their target genes.^[^ ^40^ ^]^ However, research on ischaemic flaps remains limited. Our results showed that inhibiting the expression of KEAP1 promoted flap survival and that ABs could deliver miRs, and we wondered whether miRs played a role in this process. Thus, miR sequencing was used to determine the expression of miRs in ABs and in flap tissues in the PBS group and ABs group on POD7. The results showed increased and decreased expression of 45 miRs in the ABs and PBS groups, respectively, and 30 of these miRs were consistent with a list of miRs carried in ABs. By comparing KEAP1‐targeting miRs with data from the online miR target databases TargetScan and Diana, it was found that mmu‐miR‐339‐5p may mediate the ability of ABs to inhibit KEAP1. miR‐339‐5p directly targeted KEAP1, as shown by RNA immunoprecipitation (RIP) and luciferase reporter assays. After comparing the effects of LV and siRNA transfection on the regulation of miR‐339‐5p in ABs, we used an LV for further miR regulation. We then designed and constructed LVs to inhibit or promote miR‐339‐5p expression in FSCT cells and finally extracted the ABs‐miR‐339‐5p in and ABs‐miR‐339‐5p oe. In vitro and in vivo experiments demonstrated that by reducing the delivery of miR‐339‐5p, ABs inhibited ferroptosis through the miR‐339‐5p/KEAP1/Nrf2 axis, thereby increasing ischaemic flap survival.

This study is the first to effectively show that FSCT cell‐derived ABs contribute to ischaemic flap survival by inhibiting ferroptosis. However, in addition to ferroptosis, ABs play a role in protecting the ischaemic flap by inhibiting apoptosis and necrosis, but the degree to which these processes are affected requires further research. Additionally, our findings support a recent study suggesting that ABs derived from neighboring cells can be phagocytosed by surrounding cells and promote their proliferation.^[^ ^9^ ^]^ FSCT‐ABs can be used as a precise treatment for patients, particularly in second‐stage clinical flap repair surgery. The relative simplicity of the preparation allows many ABs to be obtained after in vitro treatment of FSCT cells from patients. These ABs can be used as independent therapeutic agents or encapsulated drugs for targeted delivery (facilitating phagocytosis due to the presence of phosphatidylserine on the membrane surface) during flap surgery, making this approach an effective strategy. Our goal is to optimize the use of ABs derived from human cells and facilitate further clinical studies involving human participants, which we are initiating. Interestingly, among EV subtypes, ABs inherit the characteristics of blast cells to the greatest extent.^[^ ^9^, ^24^, ^41^ ^]^ Considering the versatility of stem cells, whether ABs produced by stem cells promote tissue repair to a greater extent than FSCT cell‐derived ABs, such as those produced from adipose stem cells or bone marrow MSCs, remains to be determined. We demonstrated that FSCT cell‐derived ABs inhibited KEAP1 by delivering miR‐339‐5p. In addition to miRs, ABs contain many proteins and other RNAs (lncRNAs and circRNAs), and therapeutic protein delivery via EVs has been frequently reported.^[^ ^42^ ^]^ However, whether the ABs in this study also inhibited ferroptosis through the delivery of certain proteins is unknown, and further analysis is needed. The exosomes of MSCs derived from human umbilical cord blood (HUCB‐MSC‐Exos) were suggested to suppress divalent metal transporter 1 (DMT1) expression via miR‐23a‐3p to inhibit ferroptosis and attenuate myocardial injury.^[^ ^43^ ^]^ We focused on the role of ABs in mediating the KEAP1/Nrf2/SLC7A11 axis to suppress ferroptosis in ischaemic flaps. We will further investigate the impact of other pathways on ferroptosis in future studies. Our findings indicate that while knocking out miR‐339‐5p may impact the therapeutic effects of ABs, it is not solely responsible for their pharmacological properties. Thus, it is imperative to further examine the roles of other pathways in mediating the effects of ABs. Moreover, in addition to the fact that different types of stem cells produce EVs that carry different signals, the pretreatment of parent cells also affects the contents of EVs.^[^ ^44^ ^]^ Recent research on exosomes extracted from hypoxic cells has attracted much attention.^[^ ^45^ ^]^ When cells are exposed to hypoxia, many anti‐hypoxia genes are expressed, and so hypo‐ABs produced by these parent cells will also contain more anti‐hypoxia genes. Whether these hypo‐ABs are suitable for the treatment of ischaemic flap tissue also requires further investigation.

In our study, ABs were shown to be useful therapeutic agents for ischaemic flaps. Most of the current research on EVs used BCA to examine and quantify them. Indeed, the number of ABs may be related to the treatment. We will further examine whether the number of ABs is related to the treatment effect in subsequent studies. Apoptotic cells can produce membrane‐encapsulated ABs with higher efficiency than exosomes produced by living cells. More importantly, compared with the extraction of exosomes, the extraction of ABs is simpler, and apoptosis can be completely controlled through standardized protocols.^[^ ^46^ ^]^ We observed the status of ABs on POD3 in vivo. It is necessary to consider longer time points to examine the effects after ABs are no longer present, such as on days 7, 14, and 21. We will also further investigate this aspect in future studies. In this study, we chose STS‐induced apoptosis to generate ABs, but the use of STS as a stimulus can also impact ABs contents. In future studies, we will also compare ABs generated through other methods, such as UV irradiation‐ and hypoxia‐induced apoptosis. A recent study demonstrated the presence of circulating ABs and revealed that they were important.^[^ ^24^ ^]^ Based on these findings, it is feasible to collect circulating ABs from a specific patient for the treatment of ischaemic skin flaps. In addition to miR‐339‐5p, other components of ABs alter specific pathways. Moreover, exosomes engineered to carry certain therapeutic proteins or noncoding RNAs have also been widely reported.^[^ ^47^ ^]^ ABs transport a variety of signaling molecules, including miRs, proteins, and circRNAs, and thereby affect multiple signaling pathways.^[^ ^9^, ^24^, ^48^ ^]^ Our primary focus was on miRs because they are present at the highest levels in ABs among all subtypes of EVs. Future research on therapeutic factors other than miR‐339‐5p will be performed. Whether ABs, which are the type of EV that contain the most parental information, are superior for the engineered delivery of therapeutic proteins or RNAs requires further research. Moreover, ABs have great application prospects in bio‐composites, regardless of whether they are bound to fibres, carried in hydrogels or contained in a drug delivery system, which is another future application direction.^[^ ^49^ ^]^ ABs also have advantages over other types of EVs because of their unique phosphatidylserine protein. More interestingly, based on the present results, ABs resist injury and promote ischaemic flap repair. Therefore, it might be feasible to perform pre‐stimulation with ABs before surgical flap transplantation, but understanding whether this can improve the resistance of the flap to damage and hypoxia and improve recovery requires further examination. The miR‐339‐5p/KEAP1/Nrf2/SLC7A11 pathway inhibits ferroptosis and provides a new target for ferroptosis inhibition. Finally, ABs act as a double‐edged sword. EVs from apoptotic cells contain fragmented DNA, which activates the cGAS signaling pathway and leads to cell death.^[^ ^50^ ^]^ Understanding whether this cell death is critical to the therapeutic effect requires further examination. Overall, we believe that the current work reveals the possible application of ABs in ischaemic flap repair and provides a new direction for future clinical treatment.

In summary, we found that ABs produced by cells in adjacent tissue promoted the recovery of damaged tissue in ischaemic flaps by reducing ROS and cell death and promoting the M1‐to‐M2 transition in macrophages. It was also confirmed that FSCT cell‐derived ABs promoted ischaemic flap survival by inhibiting ferroptosis in endothelial cells and macrophages. Furthermore, the results suggested that ABs delivered miR‐339‐5p to inhibit KEAP1 expression, thereby increasing the level of nuclear Nrf2 in endothelial cells and macrophages. Overall, FSCT cell‐derived ABs inhibited ferroptosis and promoted the M1‐to‐M2 transition in macrophages through the miR‐339‐5p/KEAP1/Nrf2 axis in ischaemic flaps and promoted ischaemic flap survival (Figure 9k). Therefore, we believe that FSCT cell‐derived ABs are beneficial for the treatment of ischaemic flap necrosis and have potential clinical application prospects and that further in‐depth exploration would be valuable. Our results also provide a new direction for ischaemic flap therapy. However, translating the use of ABs into the clinic requires further evaluation and optimization.

## 4 Experimental Section

### Cells

L‐Wnt‐3A (FSCT cells), Raw264.7 and bEnd.3 cells were obtained from Procell Life Science & Technology Co., Ltd. The cells were cultured in an incubator with 5% CO_2_ and 95% air at 37 °C. DMEM (Procell, PM150210) supplemented with sterile 10% FBS and 1% penicillin‒streptomycin. bEnd.3 cells were treated with TBHP solution (IC50: 110 µm) for 1 day to mimic oxidative stress damage. Cell division was confirmed in the NC, TBHP, TBHP + ABs, TBHP + ABs‐Scramble, TBHP+ABs‐miR‐339‐5p in and TBHP+ABs‐miR‐339‐5p oe groups. FSCT cells were treated with PBS, LV‐miR‐NC, LV‐miR‐339‐5p in or LV‐miR‐339‐5p oe before ABs extraction. siRNA was used to inhibit miR‐339‐5p after ABs extraction. LV‐miR‐NC, LV‐miR‐339‐5p in, LV‐miR‐339‐5p oe and siRNA‐339‐5p were obtained from GeneChem Chemical Technology.

### Isolation and Characterization of ABs

FSCT cells were incubated with 0.5 µmol L^−1^ STS (MedChemExpress, Shanghai, China) at 37 °C in 5% CO_2_ to induce apoptosis. Annexin V‐FITC/PI staining was used to determine apoptosis in FSCT cells treated with STS. Fifteen hours later, the cell supernatants were centrifuged for 5 min to remove cell debris. The supernatants were then centrifuged for 30 min at 2000 × g. ABs derived from FSCT cells were resuspended in PBS for subsequent use (three times) (Figure 1a). A BCA protein assay was used to determine the concentration of FSCT cell‐derived ABs. The size distribution of the ABs (gated size with mouse platelets) was measured using FCM. WB was performed to identify ABs based on surface marker proteins (H3, H2B, C1QC, C3B and CASP‐3), and *β*‐actin served as an internal control. Annexin V‐FITC/PI staining was used to examine phosphatidylserine (PS), a specific indicator that is highly expressed in ABs.

### Internalization of ABs into ECs and Macrophages In Vitro

bEnd.3 and Raw264.7 cells in dishes were plated and maintained them at 37 °C for one night. ABs were labeled with DiI from a cell plasma membrane staining kit (Biotechnology, C1991S) according to the manufacturer's instructions and used PBS for the wash steps (three times). The washed ABs were centrifuged for 30 min at 2000 × g. Then, 15 µg mL^−1^ DiI‐labelled ABs were cocultured with bEnd.3 cells for 24 h. After 15 min of fixation with 4% paraformaldehyde (Solarbio, P1110) at 4 °C, the cell nuclei were counterstained with Hoechst 33342 (Biosharp Life Sciences, BL803A). Trypsin‐eluted cells were resuspended in PBS and centrifuged three times before FCM.

### Animals

The animal experiments performed in this study conformed to the Guidelines for the Welfare and Usage of Lab Animals of the China National Institutes of Health and were approved by the Animal Welfare and Use Committee of Wenzhou Medical University (wydw2024‐0057). C57BL/6J mice (male, 6–8 weeks, mean bodyweight of 20–30 g) were obtained from the Experimental Animal Center of Wenzhou Medical University (no. SCXK [ZJ] 2015‐0001). The mice were kept under normal conditions (21–25 °C, 50–60% humidity, 12‐h light/dark cycle) and had free access to food and water.

### FLAP Modelling

The animals were anaesthetized by an intraperitoneal injection of 1% (w/v) sodium pentobarbital. Then, an electric shaver and depilatory cream (Veet, Reckitt Benckiser, UK) were used to remove the fur from the backs of the anaesthetized mice with a random flap generated as previously described. The caudal skin/sarcoma flap (dimensions: 1.5 × 4.5 cm^2^) was lifted under the dorsal fascia with sterile instruments. Subsequently, the left and right sacral arteries supporting the blood supply to the flap were completely resected. Mice in the PBS group and ABs group were subcutaneously injected with 100 µL (eight injection sites) of PBS and ABs (1 mg mL^−1^) via a microinjection needle along the immediate extension axis. Finally, the separated flap was directly inserted into the donor bed and sutured with 4‐0 nonabsorbable silk sutures (Figure S2a, Supporting Information). Penicillin (30000 U kg^−1^) was intramuscularly injected daily after surgery to prevent infection. Carprofen (25 mg kg^−1^, oral, with water, every day after surgery) was used for postoperative analgesia. Then, a thermal camera (FLIR One Pro, FLIR Systems, Inc., USA) was used to monitor body temperature daily to ensure that it remained at 37 ± 1 °C after surgery, indicating that no wound infection had occurred. The wound was disinfected with 1% iodophor (two times) every day after the operation to prevent postoperative infection, remove the smell of blood from the wound, and prevent bites. Each group of five mice was housed in a cage maintained at a suitable temperature and humidity with adequate food and water. The FLAP was partitioned into three areas: area I refers to the proximal region with survival characteristics, area II denotes the area at the boundary of viable necrosis, and area III corresponds to the distal region where necrosis occurs. Area II of the flap was used as the source of tissue for follow‐up experiments.

### Groups and Treatments

To overexpress KEAP1 in mouse skin flap tissue, the mice received a tail vein injection of KEAP1‐expressing adeno‐associated virus (AAV‐KEAP1) two weeks before surgery. The scrambled Control group received an identical volume of AAV that expressed a negative control sequence (AAV‐Scramble). Except for Cytokine chip detection (n = 4), RNA sequencing (n = 4) and PCR (n = 5), all of the remaining experiments used the same group size (n = 6). The C57BL/6J mice were randomly divided into 22 treatment groups: POD0 (n = 6), POD1 (n = 6), POD3 (n = 6), POD7 (n = 6), POD14 (n = 6), Control (n = 22), PBS (n = 37), ABs (n = 37), Fer‐1 (n = 12), ABs+Fer‐1 (n = 12), Erastin (n = 18), ABs+Erastin (n = 18), CL‐PBS (n = 6), CL‐FVUA (n = 6), CL‐FVUA+Fer‐1 (n = 6), ABs+Scramble (n = 24), AAV‐KEAP1 (n = 6), ABs+AAV‐KEAP1 (n = 24), ABs‐Scramble (n = 29), ABs‐siRNA‐miR‐339‐5p (n = 6), ABs‐miR‐339‐5p in (n = 29) and ABs‐miR‐339‐5p oe (n = 29). Daily i.p. injections of erastin (25 mg kg^−1^) or Fer‐1 (5 mg kg^−1^) were applied.

### Materials

The following chemicals were used in this study: dihydroethidium (cat# S0063), a cell plasma membrane staining kit with DiI (cat# C1991S), crystal volet staining solution (cat# C0121), an enhanced mitochondrial membrane potential assay kit with JC‐1 (cat# C2003S) and calcein AM (cat# C2012) from Beyotime Biotechnology (Jiangsu, China); penicillin G (C_16_H_17_KN_2_O_4_S, HPLC ≥ 98.0%, cat# P102194) and carprofen (C_15_H_12_ClNO_2_, HPLC ≥ 98.0%, cat# C153919) from Aladdin (Shanghai, China); and erastin (C_30_H_31_ClN4O4, purity≥99.76%, cat# HY‐15763) and a CCK‐8 kit (cat# HY‐K0301) from MedChemExpress (Monmouth Junction, NJ, USA). A Prussian blue iron staining kit (with Nuclear Fast Red) (cat# G1422), micro MDA assay kit (cat# BC0025) and micro reduced GSH assay kit (cat# BC1175) were acquired from Solarbio Science & Technology. A BCA assay kit (cat# 23227) and NE‐PER nucleus and cytoplasm abstraction reagents (cat# 78835) were obtained from Thermo Fisher Scientific (Rockford, IL, USA). Mounting medium with DAPI–aqueous and Fluoroshield (cat# 324 ab104139) were obtained from Abcam (Cambridge, UK). Ferro Orange (cat# F374) was purchased from Dojindo Laboratories (Japan). A Co‐IP kit (cat# Bes3011) and RIP kit (cat# Bes5101) were purchased from Bersin Bio (Guangzhou, China). Clodronate liposomes were purchased from Vrije Universiteit Amsterdam (CL‐FVUA), and clodronate liposome‐PBS (CL‐PBS) was purchased from Yesen (Shanghai, China). Primary antibodies against iNOS (cat# 13120S) and Arg1 (cat# 93668S) were purchased from Cell Signaling Technology (Beverly, MA, USA). Antibodies against KEAP1 (cat# 10503‐2‐AP), SLC7A11/xCT (cat# 26864‐1‐AP), TXNRD1 (cat# 11117‐1‐AP), GPX4 (cat# 67763‐1‐Ig), NOX1 (cat# 17772‐1‐AP), COX2/cyclooxygenase 2/PTGS2 (COX2, cat# 12375‐1‐AP), AGO2 (cat# 67934‐1‐Ig) and histone H3 (cat# 17168‐1‐AP) were obtained from the Protein Technology Group (Chicago, IL, USA). Antibodies against *β*‐actin (cat# ab213262), 4‐HNE (cat# ab48506), rabbit IgG‐H&L DyLight 488 (cat# ab96883), goat anti‐mouse IgG‐H&L DyLight 488 (cat# ab96871), and goat anti‐mouse IgG‐H&L DyLight 594 (cat# ab96873) were purchased from Abcam (Cambridge, UK). Antibodies against histone H2B (cat# A1958), C1QC (cat# A9227) and complement C3 (cat# A13283) were purchased from ABclonal Technology (Cambridge, MA, USA). Antibodies against CD31 (cat# GB12063) and endomucin (cat# GB112648) were purchased from Servicebio (Wuhan, China). AAV‐EKAP1 *(NM_016679)*, LV‐mmu‐miR‐339‐5p‐inhibition, LV‐mmu‐miR‐339‐5p‐overexpression and siRNA‐mmu‐miR‐339‐5p were acquired from Shanghai GeneChem Co. Ltd. (Shanghai, China).

### Infrared Thermal Imaging

The FLIR One Pro external probe for infrared thermal imaging and a mobile phone were used to capture thermal images of the ischaemic flaps.^[^ ^51^ ^]^ The average temperatures of the surgical area (FLAP) and the head and neck area were measured separately (normal skin). The Delta‐T was determined as the difference between the FLAP temperature and normal skin temperature. The differences in temperature between the groups were analyzed and compared. The better the flap recovered, the closer the delta‐T was to 0 °C. To mitigate the impact of substantial variations in basal body temperature among the mice on the results, the basal body temperatures were analyzed across the groups. The results revealed no statistically significant differences between the groups.

### LDBF Analysis

LDBF analysis was used to visualize the vascular network of the FLAP. After anaesthesia on POD7, the mouse was kept in a safe environment without disturbances. Then, a laser Doppler instrument was used to assess the blood supply to the skin flap. LDBF analysis was performed as previously described. moorLDI Review software was used to calculate the perfusion units (PUs), and the blood flow was evaluated (ver. 6.1; Moor Instruments). Blood flow was measured in each mouse three times and used the average value for statistical analysis.

### Immunohistochemistry

Mouse FLAP tissue was fixed with 4% paraformaldehyde (Solarbio, P1110). The paraffin‐embedded FLAP tissue was cut from area II into 4‐µm sections after dehydration. Xylene was used to deparaffinize the sections. The deparaffinized tissue first underwent rehydration, followed by antigen retrieval in sodium citrate buffer. The sections were cooled to room temperature and blocked with 10% goat serum (Beyotime, C0265) in PBS (Procell, PB180327) containing 0.1% Triton X‐100 (Aladdin, T109027). Then, the sections were incubated overnight with primary antibodies at 4 °C and for 1 h with secondary antibodies at room temperature the next day. Nuclei were stained with DAPI. The primary antibodies used targeted CD31 (1:200), EMCN (1:200), CD68 (1:200), iNOS (1:200), Arg1 (1:200), SLC7A11 (1:200) and GPX4 (1:200). The following goat polyclonal secondary antibodies were used: rabbit IgG‐H&L DyLight‐488 (Abcam, ab96883), goat anti‐mouse IgG H&L DyLight‐488 (Abcam, ab96871), goat anti‐mouse IgG‐H&L DyLight‐594 (Abcam, ab96873), and goat anti‐rabbit IgG‐H&L DyLight‐594 (Abcam, ab96885). An in situ cell death examination kit was used to perform TUNEL assays on frozen skin sections according to the manufacturer's protocol. Frozen skin sections were stained with DHE (Beyotime, S0063) according to the manufacturer's protocol. A Masson's Trichrome Stain Kit was used to examine collagen in the skin (Solarbio, G1340).

### Flow Cytometry

According to relevant literature,^[^ ^52^ ^]^ single cell suspensions of skin flap tissue was extracted for the next step of flow cytometry staining among three groups. Referring to the flow cytometry staining technology,^[^ ^53^ ^]^ use Anti‐Mouse F4/80, PE (Clone: BM8.1; cat# F21480A01‐25, MutiScience), Anti‐Mouse CD80, APC (Clone: 009; cat# F21080A03‐25, MutiScience), and FITC anti‐mouse CD206 (MMR; cat# 141703, BioLegend) FCM primary antibodies for tissue cell staining and on‐machine detection, and FlowJo performs flow cytometry data analysis.

### Antibody Array

As previously described,^[^ ^54^ ^]^ the Mouse Cytokine Array GS4000 (RayBiotech, GSM‐CAA‐4000‐1) was used to extract proteins from the Control group, the PBS group and the ABs group to examine 200 cytokines on the chip.

### WB

Dissection and homogenization of flap tissue samples from area II were performed in ice‐cold RIPA lysis buffer (Beyotime, P0013B) supplemented with phenylmethanesulfonylfluoride (PMSF; Beyotime, ST506) and protease and phosphatase inhibitor cocktails (Beyotime, P1046). The homogenates were centrifuged for 30 min at 20 000 ×g and 4 °C to collect the tissue lysate. An Omni‐Easy Instant BCA protein assay kit (Epizyme, ZJ102 L) was used to measure the protein concentration. Samples containing 30 µg of protein were loaded on 4–22% SDS‒PAGE gels, and the proteins were then transferred onto PVDF membranes (Millipore). The membranes were blocked with 5% skim milk (BD Biosciences, 232, 100), and primary antibodies were added and incubated for 15 h at 4 °C; then, the membranes were incubated for 1.5 h with HRP‐conjugated secondary antibodies at room temperature. An Omni‐ECL Pico Light Chemiluminescence Kit (Epizyme, SQ201) was used to examine the protein bands, and a ChemiDoc system (Bio‐Rad) was used for visualization. Image Lab software (Bio‐Rad) was used to analyze the bands. Primary antibodies against the proteins SLC7A11 (1:1000), Txnrd1 (1:1000), GPX4 (1:1000), ACSL4 (1:1000), HO‐1 (1:1000), COX2 (1:1000), NOX1 (1:1000), CASP‐3 (1:1000), *β*‐Actin (1:1000), Histone H3 (1:1000), KEAP1 (1:1000), Nrf2 (1:1000), H2B (1:1000), C1QC (1:1000), and C3B (1:1000) were used. The secondary antibodies used were HRP‐conjugated AffiniPure goat anti‐mouse IgG (H+L) (Proteintech, SA00001‐1) and HRP‐conjugated AffiniPure goat anti‐rabbit IgG (H+L) (Proteintech, SA00001‐2).

### ELISA

The activity of HMGB1 (Qzkndbio, SU‐B20866), 8‐OHdG (Qzkndbio, SU‐B20120), TNF‐*α* (Qzkndbio, SU‐B20220), IFN‐*β* (Qzkndbio, SU‐B20334), COX2 (Qzkndbio, SU‐B20860), LPO (Qzkndbio, SU‐B21196) and NOX1 (Qzkndbio, SU‐B21140) were assessed using ELISA kits according to the manufacturer's instructions. Optical density readings to quantify HMGB1, 8‐OHdG, TNF‐α, IFN‐β, COX2, LPO and NOX1 were obtained with a microplate reader at 550 nm using a correction wavelength of 450 nm.

### RNA Isolation and Library Preparation

TRIzol reagent was used for total RNA extraction according to the manufacturer's protocol. A NanoDrop 2000 spectrophotometer was used to evaluate RNA purity and concentration (Thermo Scientific, USA). An Agilent 2100 bioanalyzer (Agilent Technologies, Santa Clara, CA, USA) was used to assess RNA integrity. Then, a TruSeq Stranded mRNA LT Sample Prep Kit was used to construct the libraries according to the manufacturer's instructions (Illumina, San Diego, CA, USA). OE Biotech (Shanghai, China) performed transcriptome sequencing and analysis.

### mRNA‐seq

The libraries were sequenced on an Illumina NovaSeq 6000 platform, and 150 bp paired‐end reads were generated. Raw reads in the fastq format were first processed using fastp, and low‐quality reads were removed to obtain the clean reads. Then, the clean reads for each sample were retained for subsequent analyses. The clean reads were mapped to the reference genome using HISAT2. The FPKM of each gene was calculated, and the read counts of each gene were obtained by HTSeq‐count. PCA was performed using R (v 3.2.0) to evaluate the biological duplicates of the samples. Based on the hypergeometric distribution, GO, KEGG pathway, Reactome and WikiPathways enrichment analyses of DEGs were performed to screen the significantly enriched terms using R (v 3.2.0). R (v 3.2.0) was used to construct the column diagram, chord diagram and bubble diagram of the significantly enriched terms.

### miR‐seq

The basic reads were converted into sequence data (also called raw data/reads) by base calling. Low‐quality reads were filtered, and the reads with 5′ primer contaminants and poly(A) were removed. The reads without 3′ adapter and insert tags and the reads shorter than 15 nt or longer than 41 nt from the raw data were filtered, and clean reads were obtained. The length distribution of the clean sequences in the reference genome was determined, and the sequences were subsequently aligned and subjected to a Bowtie search against Rfam v.10.1 (http://www.sanger.ac.uk/software/Rfam); rRNA, scRNA, Cis‐reg, snRNA, tRNA and other RNAs were annotated and filtered. Then, the cDNA sequence and Repbase database of the species repeat sequence were identified with Bowtie software. The mature miRs were identified by alignment against the miRBase v22 database (http://www.mirbase.org/), and the expression patterns in the different samples were analyzed. Differentially expressed miRs were identified and filtered with the following thresholds: q value < 0.05 and FC > 2 or FC < 0.5. The q value was calculated with the DEG algorithm.

### Assays to Measure the Tissue Iron, GSH and MDA Levels

A tissue iron levels assay kit was used to test the iron levels in area II of FLAP tissue (Solarbio, BC4355). MDA levels in bEnd.3 cells and in area II of FLAP tissue were examined by a micro MDA assay kit (Solarbio, BC0025). GSH levels in bEnd.3 cells and area II FLAP tissue were evaluated using a micro‐reduced GSH assay kit (Solarbio, BC1175).

### qPCR

Total RNA was extracted as previously described. Quantitation was completed via a two‐step reaction: reverse transcription (RT) and PCR. Every RT process involved 0.5 µg of RNA, 2 µL of 5 × TransScript All‐in‐One SuperMix for qPCR and 0.5 µL of gDNA Remover (10 µL). The reaction was performed using a GeneAmp PCR System 9700 (Applied Biosystems, USA) for 15 min at 42 °C and then for 5 s at 85 °C. Subsequently, the 10‐µL RT reaction mixture was desaturated ten times at −20 °C in nuclease‐free water. Real‐time PCR was performed using a Light Cycler 480II real‐time PCR instrument (Roche, Switzerland) with 10 µL of PCR mix, 1 µL of cDNA, 5 µL of 2 × PerfectStart Green qPCR SuperMix (TransGen Biotech Co., AQ601), 0.2 µL of forward primer, 0.2 µL of reverse primer and 3.6 µL of nuclease‐free water. The reaction was performed in a 384‐well optic plate for 0.5 min (Roche, 04729749001) at 94 °C and then for 45 cycles of 5 s at 94 °C and 30 s at 60 °C. The specimens were analyzed three times. After the PCR cycles were complete, a melting curve assay was used to verify the production of the anticipated PCR products. The following primer sequences were synthesized by GeneChem using the mRNA sequences acquired from the NCBI database: *mmu‐miR‐339‐5p 5′‐ CGGGCTCCCTGTCCTCCAGGA* −*3′* (forward) and *5′‐ TTCTCCTTAATGTCACGCACGATT* −*3′* (reverse); *U6 5′‐ CTCGCTTCGGCAGCACA* −*3′* (forward) and *5′‐ AACGCTTCACGAATTTGCGT* −*3′* (reverse); *KEAP1 5′‐ TCGAAGGCATCCACCCTAAG* −*3′* (forward) and *5′‐ CTCGAACCACGCTGTCAATCT* −*3′* (reverse); *Actb 5′‐ CTACCTCATGAAGATCCTCACCGA* −*3′* (forward) and *5′‐ TTCTCCTTAATGTCACGCACGATT* −*3′* (reverse); and mmu‐miR‐339‐5p stem‒loop sequence: *5′‐ CCTGTTGTCTCCAGCCACAAAAGAGCACAATATTTCAGGAGACAACAGGCGTGAGC* −*3′*. The expression of the target mRNAs and target miRs were normalized to *Actb* mRNA expression and *U6* mRNA expression, respectively. The 2‐ΔΔCt method was used for qPCR analyses.

### RIP Assay

293T cells overexpressing miR‐339‐5p or miR‐scramble were collected by centrifugation and lysed in cell lysis buffer for WB and IP (Beyotime, P0013). Antibody‐conjugated beads specific for each IP reaction (Bersin Bio, Bes5101) were resuspended in RIP buffer (900 µL; Bersin Bio, Bes5101) and then added to the lysate (100 µL). The IP reactions were incubated overnight with shaking at 4 °C, and then, RIP wash buffer was used to wash the samples three times. Proteinase K (Sigma–Aldrich, 1245680100) digestion and TRIzol reagent were used to extract the immunoprecipitated RNA. qPCR was used to quantify the mRNA.

### Co‐IP Assay

Area II of the FLAPs in the PBS group and the ABs group were minced and lysed using cell lysis buffer for WB and IP (Beyotime, P0013). For each IP experiment, antibody‐coupled beads (Bersin Bio, Bes3011) were resuspended in 900 µL of primary anti‐KEAP1 antibody (1:50) and added to the lysate (100 µL). The IP mixtures were incubated overnight with shaking at 4 °C, followed by three washes with Co‐IP wash buffer. The loading buffer was boiled to release and extract the immunoprecipitated proteins. The KEAP1 and Nrf2 proteins were quantified by WB.

### Luciferase Reporter Assay

The 3ʹ‐UTR of *KEAP1* mRNA containing WT or MUT mmu‐miR‐339‐5p‐binding sequences was synthesized by Duolaimi Biotechnology (Wuhan, China). These sequences were cloned and inserted into a pmirGLO luciferase control reporter vector (Promega, E1330) to generate KEAP1 3′‐UTR reporter constructs (i.e., *pmirGLO‐WT‐KEAP1* and *pmirGLO‐MUT‐KEAP1*). Cultured 293T cells were transfected with a miR mimic (mmu‐miR‐339‐5p) or negative control (miR‐NC) at 2 × 10^4^/well in 24‐well culture plates and then applied 2 µg of *pmirGLO‐WT‐KEAP1* or *pmirGLO‐MUT‐KEAP1* for cotransfection. Five hours later, the transfection medium was removed and replaced it with complete DMEM (HyClone, SH30022.01) containing 10% FBS (LONSERA, A511‐001). A dual‐luciferase reporter gene assay kit was used to measure luciferase activity two days after transfection (Beyotime, RG027).

### Cell Counting Kit 8 (CCK‐8) Assay

bEnd.3 cells were seeded in 96‐well plates at a concentration of 9 × 10^3^ cells per well. Each well had a cell density of 50%; the cells were treated with various concentrations (0, 7.5, 15, 30, 60, 120, 240 and 480 µm) of a TBHP solution (MACKLIN, 70% in H_2_O, C_4_H_10_O_2,_ B802372). After one day of incubation, the medium containing TBHP was discarded and replaced it with fresh cell culture medium (90 µL) after the cells were washed with PBS. Then, 10 µL of CCK‐8 solution was added to each well, followed by 3 h of incubation at 37 °C. A microplate reader was used to measure the absorbance of each well at 450 nm.

### Immunocytochemistry

bEnd.3 cells were cultured on glass coverslips and fixed for 30 min in 4% paraformaldehyde (Solarbio, P1110), followed by 5 min of permeabilization with 0.1% Triton X‐100 in PBS (Procell, PB180327) and 30 min of blocking with 10% goat serum (Beyotime, C0265) in PBS. The cover slides were incubated overnight with primary antibodies and for 1 h with secondary antibodies in blocking solution at 37 °C. Nuclei were stained with DAPI. The primary antibodies used targeted SLC7A11 (1:200), GPX4 (1:200), 4‐HNE (1:200), KEAP1 (1:200), Nrf2 (1:200) and p‐MLKL (Affinity, AF3902). The secondary antibodies used were goat anti‐mouse IgG H&L (DyLight 594 or 488) and goat anti‐rabbit IgG H&L (DyLight 594 or 488). JC‐1 (Beyotime Biotechnology, C2003S) was used to examine the mitochondrial membrane potential in bEnd.3 cells according to the manufacturer's protocol. Ferro Orange (Dojindo Laboratories, F374) was used to examine Fe^2+^ levels in bEnd.3 cells according to the manufacturer's protocol. DHE (Beyotime Biotechnology, S0063) was used to examine intracellular ROS levels in bEnd.3 cells according to the manufacturer's protocol.

### Macrophage Polarization Assay

RAW264.7 cells in the various groups were gently detached with trypsin, resuspended, and chilled at 4 °C. Subsequently, they were fixed in 70% alcohol for 2 h. After two rounds of resuspension in PBS and adjustment to a concentration of 2 × 10^6^ cells per EP tube, the cells were incubated for 30 min with antibodies against Arg1 (1:50) and iNOS (1:50). The cells were then resuspended twice in PBS, exposed to secondary antibodies, and thoroughly washed with PBS prior to flow cytometric analysis.

### Tube Formation Assays

The angiogenic activity of bEnd.3 cells was evaluated on µ‐slides (Ibidi, 81506) precoated with growth factor‐reduced Matrigel (10 µL per well, Corning, 356234). In brief, bEnd.3 cells were stained with a cell‐permeable dye (calcein AM; Beyotime, C2012) for 40 min and then replated on Ibidi µ‐slides. After the cells were incubated for 8 h at 37 °C, computer‐assisted microscopy was used to examine the formation of capillary‐like tubes. Each tube had a tube‐like structure with a length that was three times the width. ImageJ software was used to determine tube lengths in duplicate wells.

### Cell Migration Assays

Cell migration assays were performed to examine the in vitro migration of bEnd.3 cells in Transwell inserts (8.0‐µm polycarbonate membrane; Corning, 3422). bEnd.3 cells were seeded into the upper chambers, which were then incubated for 10 h at 37 °C. The cells in each chamber were fixed in 4% paraformaldehyde (Solarbio, P1110), followed by crystal violet staining (Beyotime, C0121). A computer‐assisted microscope was used to image the migrated cells.

### Scratch Assays

A 1‐mL pipette tip was used to make a scratch in the middle of overgrown bEnd.3 cells in a six‐well plate, and the state of the scratch was observed and recorded under an inverted microscope. The bEnd.3 cells were incubated for 1 day at 37 °C. Then, the cells that had migrated to the scratch were observed with an inverted microscope. Statistical analysis of the area of cell migration was performed with ImageJ software.

### Statistical analysis

Statistical analysis was performed with the program SPSS 22 (USA). The data are shown as the average ± SEM. The data shown herein were normalized to the control data to avoid unwanted variation. ANOVA followed by LSD (equal variances assumed) post hoc analyses or Dunnett's T3 (equal variances not assumed) test was performed to assess differences among 3, 4 or 5 groups. Independent‐specimen t tests were used to assess differences between groups. P < 0.05 indicated statistical significance.

## Conflict of Interest

The authors declare no conflict of interest.

## Author Contributions

G.Y., Y.C., and N.Y. contributed equally to this work. G.Y., X.W., J.D., J.X., and K.Z. designed research. G.Y., Y.C., and N.Y. performed research. G.Y., H.Z., X.Z., Y.G., J.Z., Z.C., C.D., L.L., J.Q., X.Z., and X.J. analyzed data. G.Y., Y.C., and N.Y. wrote the paper. X.W., W.G., Y.C., J.D., J.X., and K.Z. revised the paper. All authors have read, approved the final manuscript.

## Supporting information

Supporting Information

## Data Availability

The data that support the findings of this study are available from the corresponding author upon reasonable request.
