# Supplementary material for: Apoptotic Bodies Derived from Fibroblast‐Like Cells in Subcutaneous Connective Tissue Inhibit Ferroptosis in Ischaemic Flaps via the miR‐339‐5p/KEAP1/Nrf2 Axis
Source: Adv Sci (Weinh). 2024 Apr 19;11(24):2307238. doi: 10.1002/advs.202307238 (PMC11200024; doi:10.1002/advs.202307238)
Supplement: Supplementary file 1 — Supporting Information [file ADVS-11-2307238-s001.pdf]

ADVANCED SCIENCE
Open Access

Supporting Information

for *Adv. Sci.*, DOI 10.1002/advs.202307238

Apoptotic Bodies Derived from Fibroblast-Like Cells in Subcutaneous Connective Tissue Inhibit Ferroptosis in Ischaemic Flaps via the miR-339-5p/KEAP1/Nrf2 Axis

Gaoxiang Yu, Yijie Chen, Ningning Yang, Haojie Zhang, Xuzi Zhang, Yibo Geng, Jiayi Zhao, Zhuliu Chen, Chengji Dong, Lidan Lin, Jianjun Qi, Xuanlong Zhang, Xiaoqiong Jiang, Weiyang Gao, Yuepiao Cai, Xiangyang Wang, Jian Ding*, Jian Xiao* and Kailiang Zhou**

Supplementary Information

For

Apoptotic Bodies Derived from Fibroblast-like Cells in Subcutaneous Connective Tissue Inhibit Ferroptosis in Ischaemic Flaps via the miR-339-5p/KEAP1/Nrf2 Axis

Gaoxiang Yu *et. al*

Figure S1

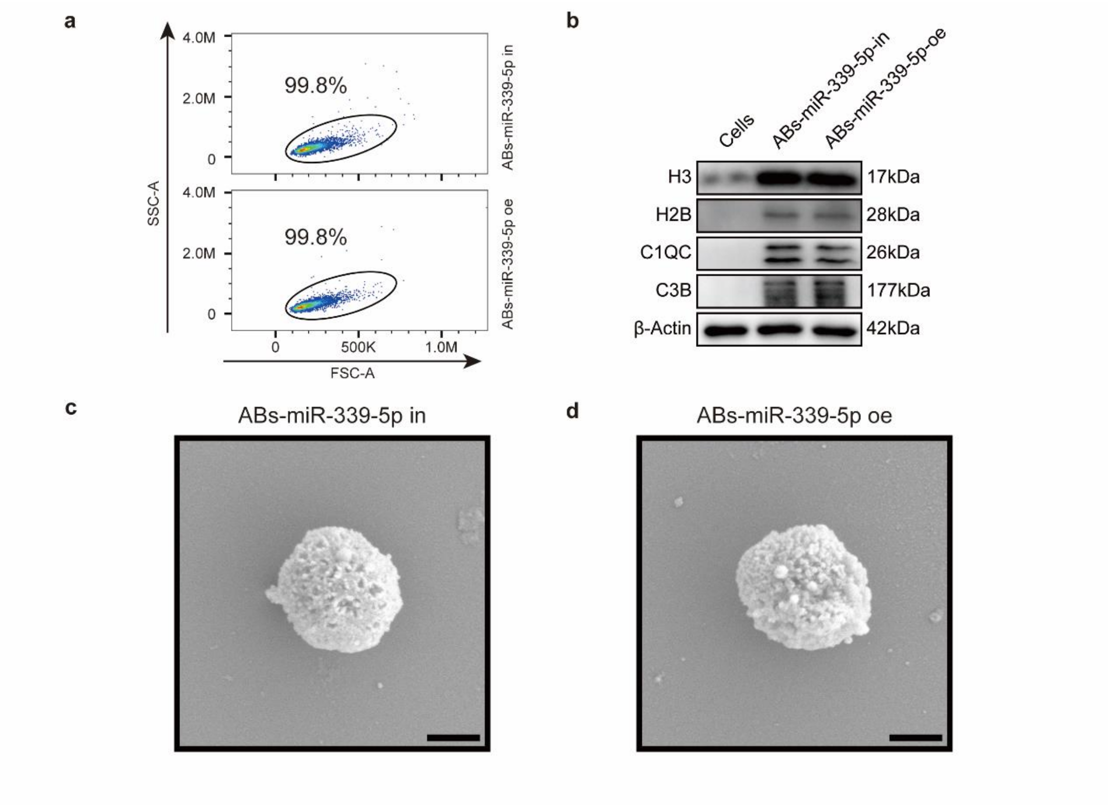

Figure S1 Characterization of AB-miR-339-5p-in and AB-miR-339-5p-oe.

a FSC/SSC analysis of AB-miR-339-5p-in and AB-miR-339-5p-oe. Frames were used as size markers (platelets, 1-4 µm) to gate ABs (Fig. 1c). **b** Western blot analysis of H3, C1QC, C3B, H2B and β-Actin in the indicated groups. **c** AB-miR-339-5p-in morphology under SEM; scale bar: 1 µm. **d** AB-miR-339-5p-oe morphology under SEM; scale bar: 1 µm.

Figure S2

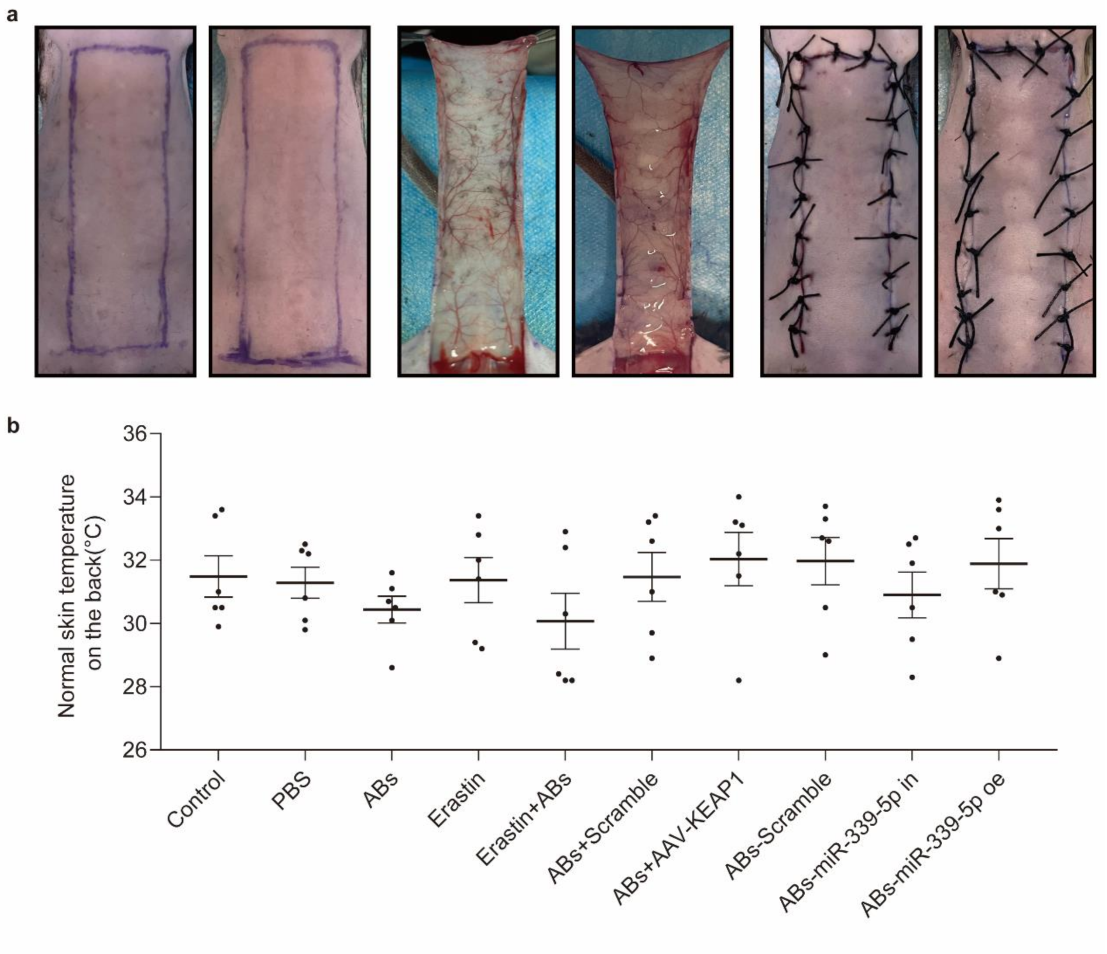

Figure S2 Demonstration of random-pattern flap modelling.

a Schematic diagram of the control and microinjection of ABs or PBS under random-pattern skin flaps. **b** Comparison of normal skin temperature on the back, head and neck in each group. The error bars are the SEMs. ANOVA was performed plus LSD post hoc analysis (equal variances) or Dunnett's T3 test (unequal variances).

Figure S3

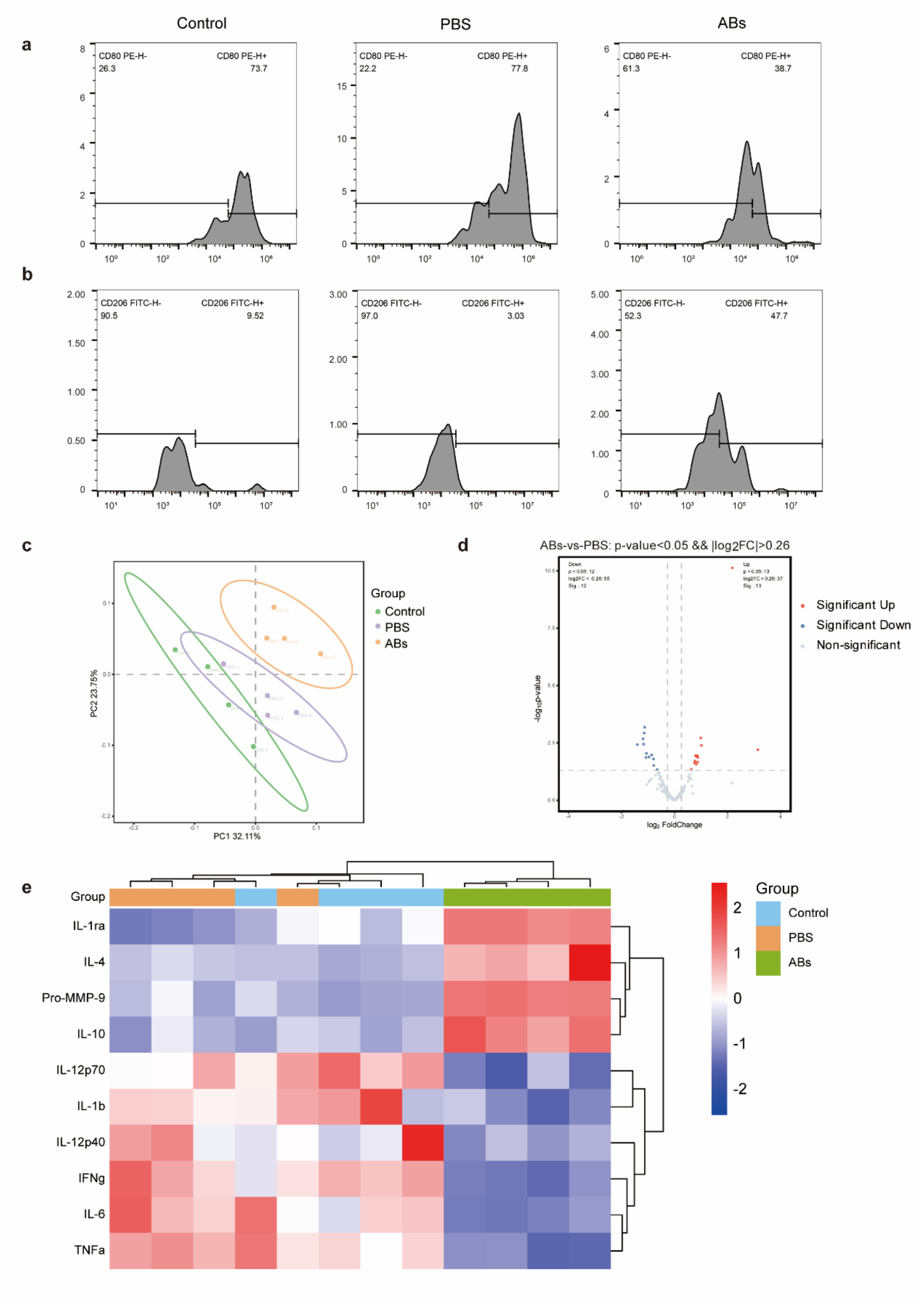

Figure S3 ABs promoted M1 macrophage to M2 polarization.

a Flow cytometric analysis of M1 macrophages (CD80) in the three groups of ischaemic skin flaps at POD7 (n = 4). **b** Flow cytometric analysis of M1 macrophages (CD206)

in the three groups of ischaemic skin flaps at POD7 (n = 4). **c** PCA of three groups of cytokine chipsets: control, PBS and ABs groups. **d** Volcano plot of cytokine chip profiles in the PBS and ABs groups. **e** Heatmap of macrophage-related differential cytokine profiles in the control, PBS and ABs groups (p *value* < 0.05).

Figure S4

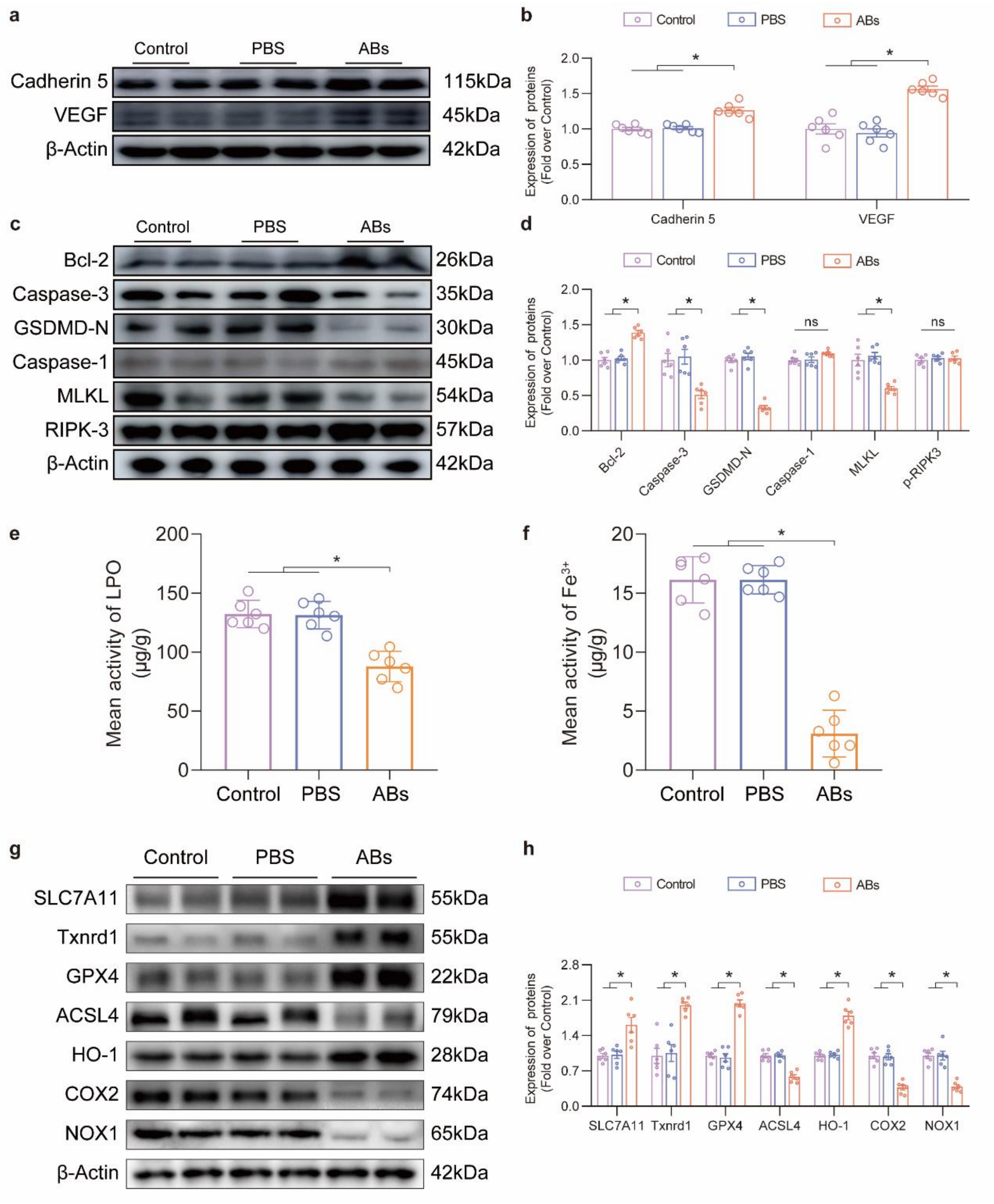

Figure S4 ABs promoted angiogenesis and inhibited cell death in ischaemic flaps. **a** Angiogenesis-related protein levels in area II of the flap in the 3 groups on POD7. **b** Quantification of angiogenesis-related proteins in the skin in the 3 groups (n = 6). **c** Apoptosis-, pyroptosis- and necroptosis-related protein levels in area II of the flap in the 3 groups on POD7. **d** Quantification of the expression of apoptosis-, pyroptosis- and necroptosis-related proteins in the skin in the 3 groups (n = 6). **e**-**f** LPO (**e**) and Fe^{3+} (**f**) levels in area II of the flap in the 3 groups (n = 6). **g** Ferroptosis-related protein levels

in area II of the flap in the 3 groups on POD7. **h** Quantification of ferroptosis-related proteins in the skin in the 3 groups (n = 6). The error bars are the SEMs. Significance (*): *p value* < 0.05; ANOVA with LSD post hoc analysis (equal variances) or Dunnett's T3 method (unequal variances).

Figure S5

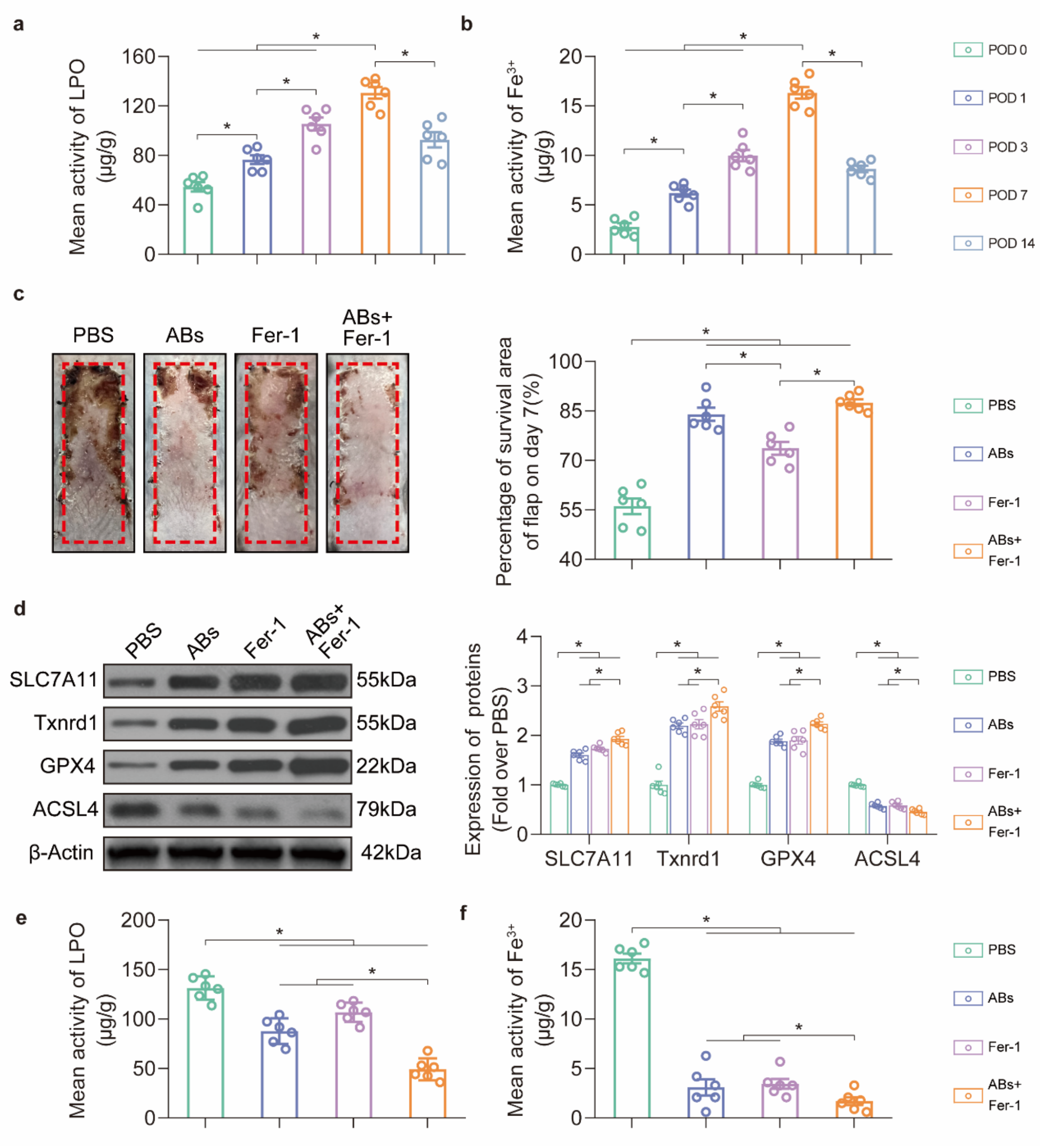

Figure S5 Targeting ferroptosis in ischaemic flaps promoted flap survival.

a-b LPO (**a**) and Fe^{3+} (**b**) levels in area II of the flap in the 5 groups (n = 6). **c** Digital image of the flap survival area on POD7. And quantification of the percentage of the survival area in the 3 groups on POD7 (n = 6). **d** Ferroptosis-related protein levels in area II of the flap in the 4 groups on POD7. And quantification of angiogenesis-related proteins in the skin in the 4 groups (n = 6). **e-f** LPO (**e**) and Fe^{3+} (**f**) levels in

area II of the flap in the 4 groups on POD7 (n = 6). The error bars are the SEMs. Significance (*): *p value* < 0.05; ANOVA with LSD post hoc analysis (equal variances) or Dunnett's T3 method (unequal variances).

Figure S6

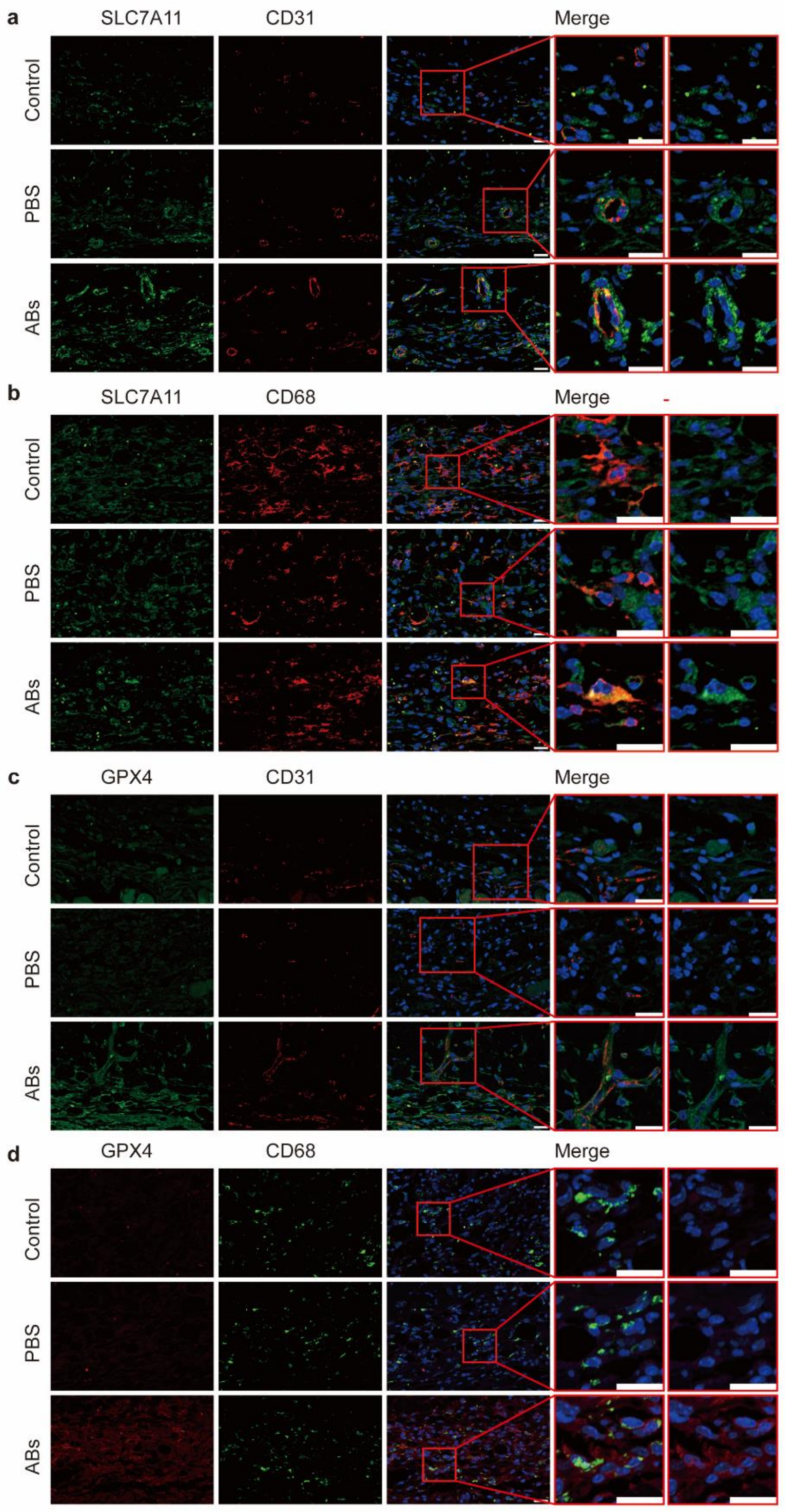

Figure S6 ABs inhibited ferroptosis in endothelial cells and macrophages in ischaemic flaps.

a Immunofluorescence costaining and magnification of CD31 and SLC7A11 in the tissue of area II of the FLAP region on POD7. Scale bars: 20 μm. **b** Immunofluorescence costaining and magnification of CD68 and SLC7A11 in the tissue of area II of the FLAP region on POD7. Scale bars: 20 μm. **c** Immunofluorescence costaining and magnification of CD31 and GPX4 in the tissue of area II of the FLAP region on POD7. Scale bars: 20 μm. **d** Immunofluorescence costaining and magnification of CD68 and GPX4 in the tissue of area II of the FLAP region on POD7. Scale bars: 20 μm.

Figure S7

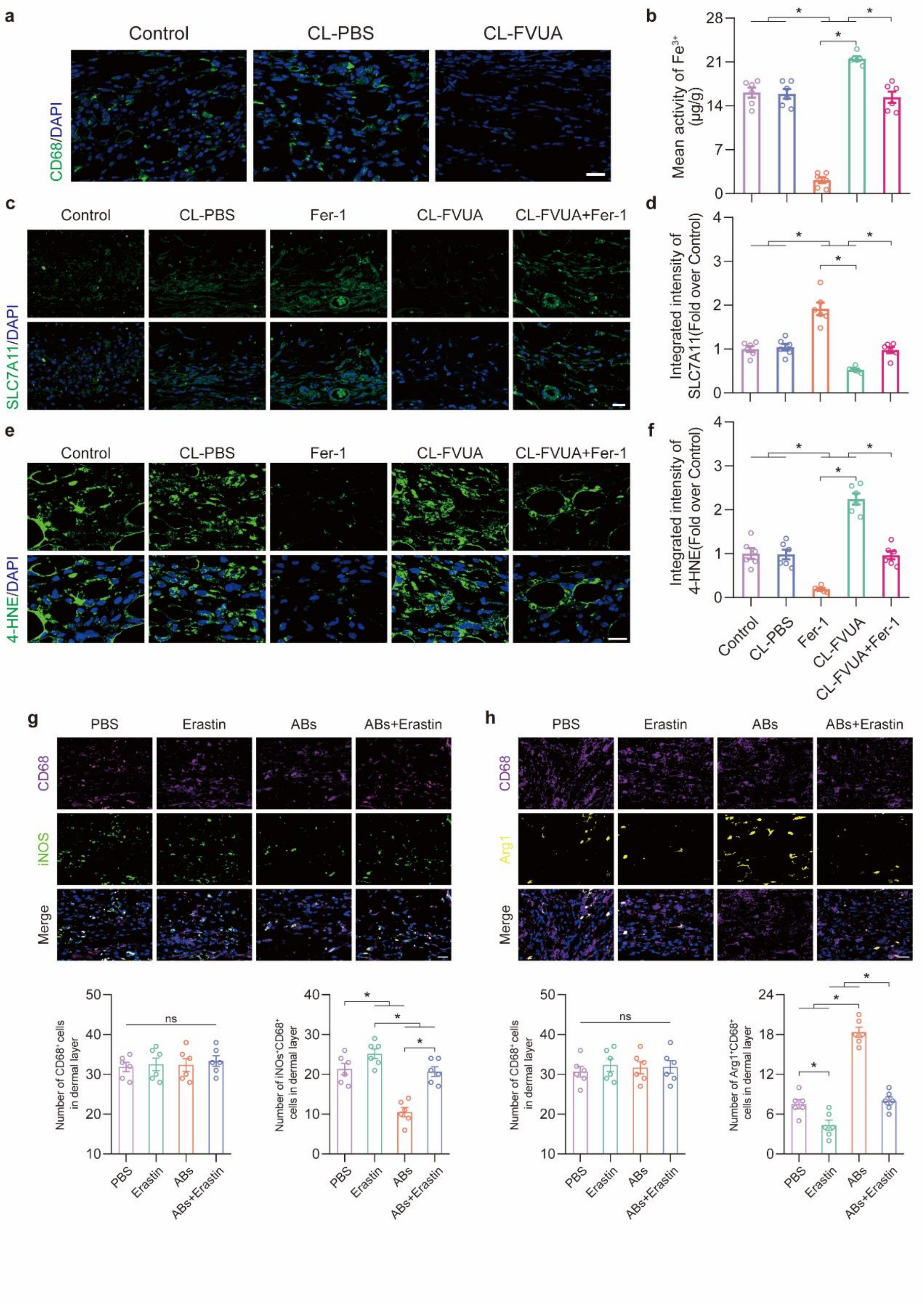

Figure S7 Crosstalk between ferroptosis and macrophage polarization in ischaemic flaps.

a IF staining of CD68 in area II of the FLAP in the 3 groups on POD7. Scale bar: 20 μm. **b** Quantification of the iron levels in area II of the FLAP in the 5 groups (n = 6). **c** IF staining of SLC7A11 in area II of the FLAP in the 5 groups on POD7. Scale bar: 20 μm. **d** Quantified integrated intensity of SLC7A11 in the dermal layer in the 5 groups (n = 6). **e** IF staining of 4-HNE in area II of the FLAP in the 5 groups on POD7. Scale bar: 20 μm. **f** Quantified integrated intensity of 4-HNE in the dermal layer in the 5 groups (n = 6). **g** CD68 and iNOS staining in area II of the flap in the 4 groups on POD7. Scale bar: 20 μm. The levels of infiltrated $CD68^+$ macrophages and M1-like ($CD68^+$ and $iNOS^+$) macrophages were quantified in the 4 groups (n=6). **h** CD68 and Arg1 staining of CD68 in area II of the flap in the 4 groups on POD7. Scale bar: 20 μm. The levels of infiltrated $CD68^+$ macrophages and M2-like ($CD68^+$ and Arg^+) macrophages were quantified in the 4 groups (n=6). The error bars are the SEMs. Significance (*): *p value* < 0.05; ns, not significant; ANOVA with LSD post hoc analysis (equal variances) or Dunnett's T3 method (unequal variances).

Figure S8

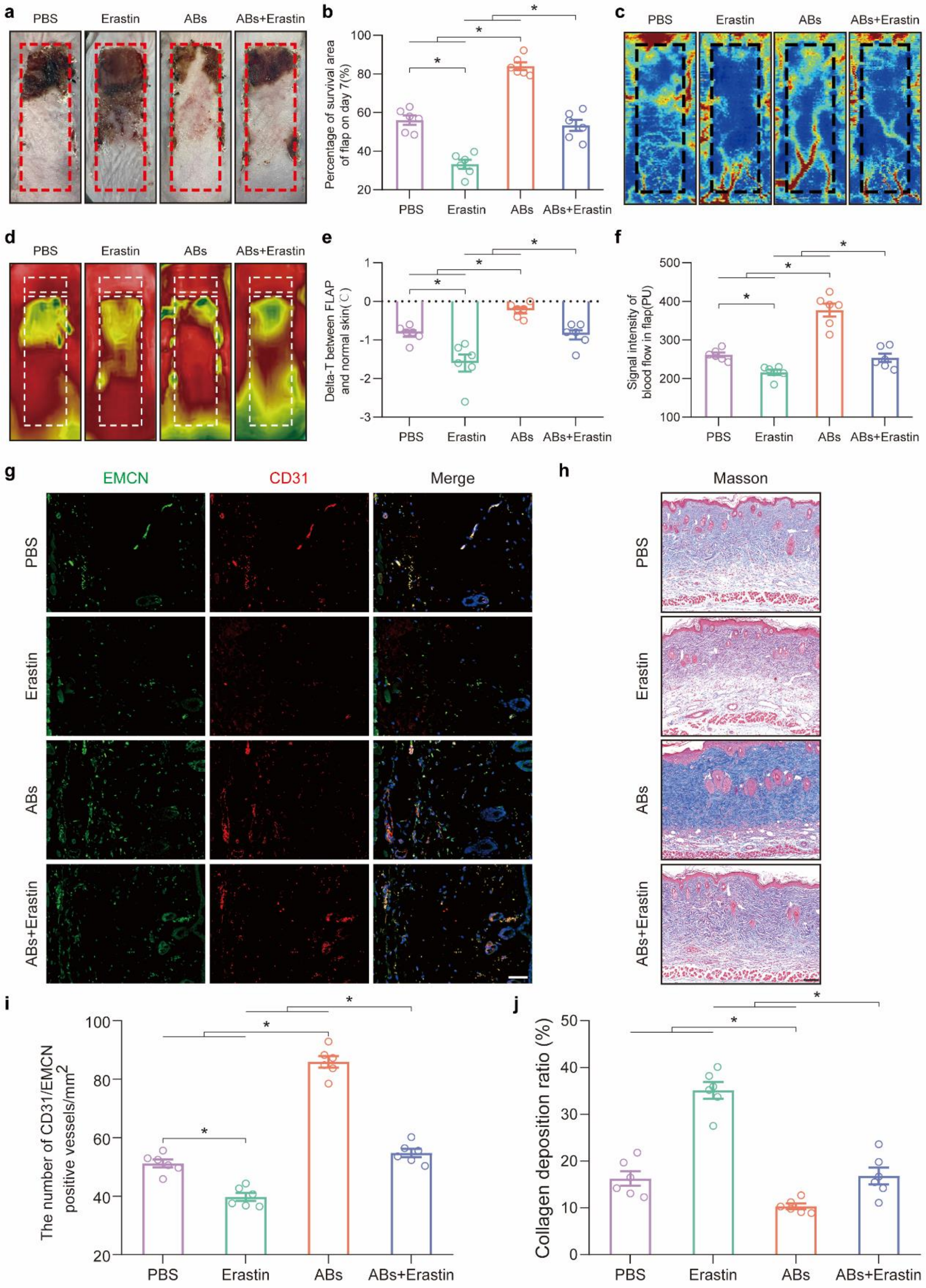

Figure S8 ABs promoted the survival of ischaemic flaps by inhibiting ferroptosis. **a** Digital photograph of the flap survival area on POD7. **b** Quantified percentage of the survival area in the 4 groups on POD7 (n = 6). **c** Images of the subcutaneous blood flow

network on POD7. **d** Thermal images of the flap on POD7. **e** Comparison of the delta-T values between FLAP and normal skin in the 4 groups on POD7. **f** Quantified blood flow signal intensity in ischaemic flaps in the 4 groups on POD7 (n = 6). **g** IF staining of CD31 and EMCN in area II of the FLAP on POD7. Scale bar: 50 μm. **h** Quantified CD31/EMCN-positive blood vessel density in the 4 groups (n=6). **i** Masson staining to examine damaged collagen in the skin on POD7. Scale bar: 100 μm. **j** Quantification of collagen deposition ratio in the 4 groups (n=6). The error bars are the SEMs. Significance (*): *p value* < 0.05; ANOVA plus LSD post hoc analysis (equal variances) or Dunnett's T3 method (unequal variances).

Figure S9

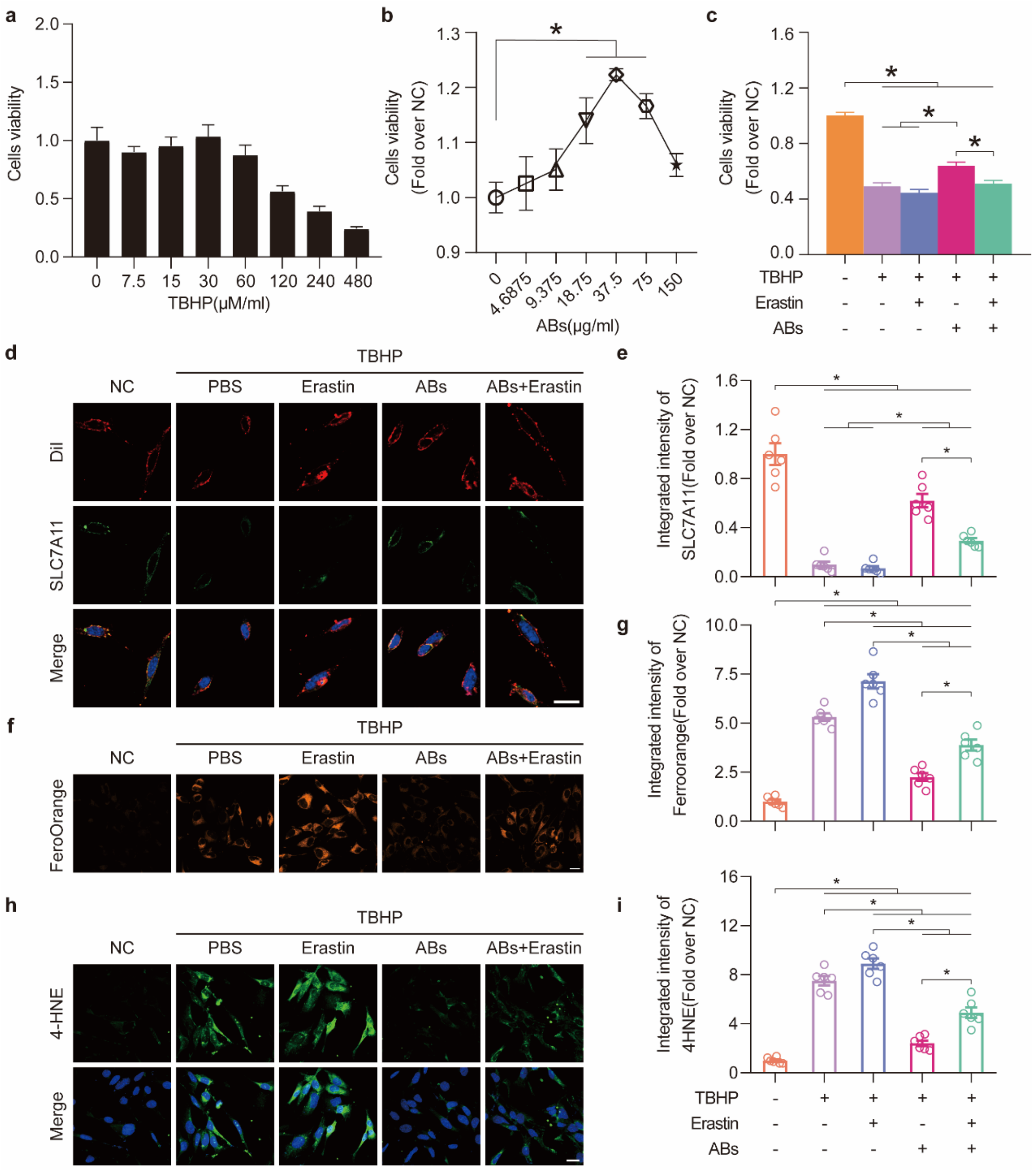

Figure S9 ABs promoted endothelial cell activity and inhibited ferroptosis in *vitro*. **a** Proliferation of bEnd.3 cells after TBHP treatment, as measured by CCK-8 assays (n = 4). **b** Proliferation of bEnd.3 cells after AB treatment, as measured by CCK-8 assays (n = 3). **c** Proliferation of bEnd.3 cells after AB treatment, as measured by CCK-8 assays (n = 3). **d** DHE staining was used to examine ROS-induced DNA damage in bEnd.3 cells in the five groups. Scale bars: 20 μm. **e** Quantification and analysis of the integral absorbance of DHE in the five groups (n = 6). **f** Cell migration assays were performed on bEnd.3 cells after 24 h in the five groups. The results were obtained after

12 h of culture. Scale bars: 50 μm. **g** Quantification and analysis of the number of migrated cells (bEnd.3 cells) (n = 6). **h** Cell scratch experiments were performed on confluent bEnd.3 cells in the five groups, and measurements were performed at 0 h and 24 h. Scale bars: 500 μm. **i** Quantification and analysis of the migration area of cells after 24 h in the five groups (n = 6). The error bars are the SEMs. Significance (*): *p value* < 0.05; ANOVA with LSD post hoc analysis (equal variances) or Dunnett's T3 method (unequal variances).

Figure S10

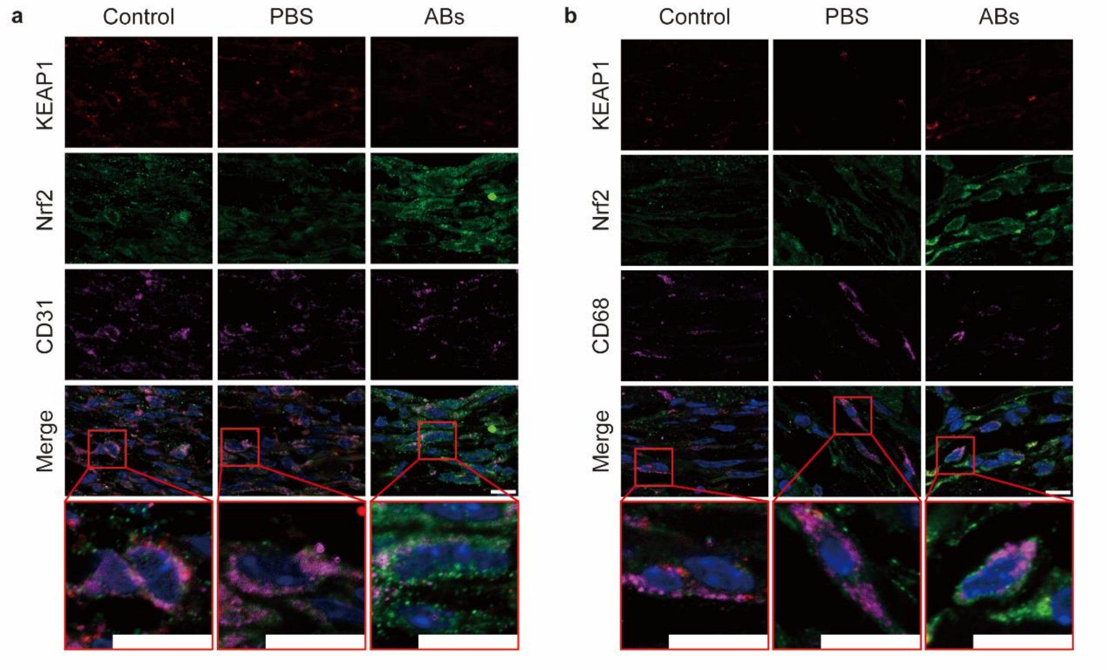

Figure S10 ABs inhibited KEAP1 and promoted nuclear Nrf2 in ischaemic flaps.
a Immunofluorescence costaining and magnification of CD31, KEAP1 and Nrf2 in the tissue of area II of the FLAP region on POD7. Scale bars: 10 µm. **b** Immunofluorescence costaining and magnification of CD68, KEAP1 and Nrf2 in tissue from area II of the FLAP region on POD7. Scale bars: 10 µm.

Figure S11

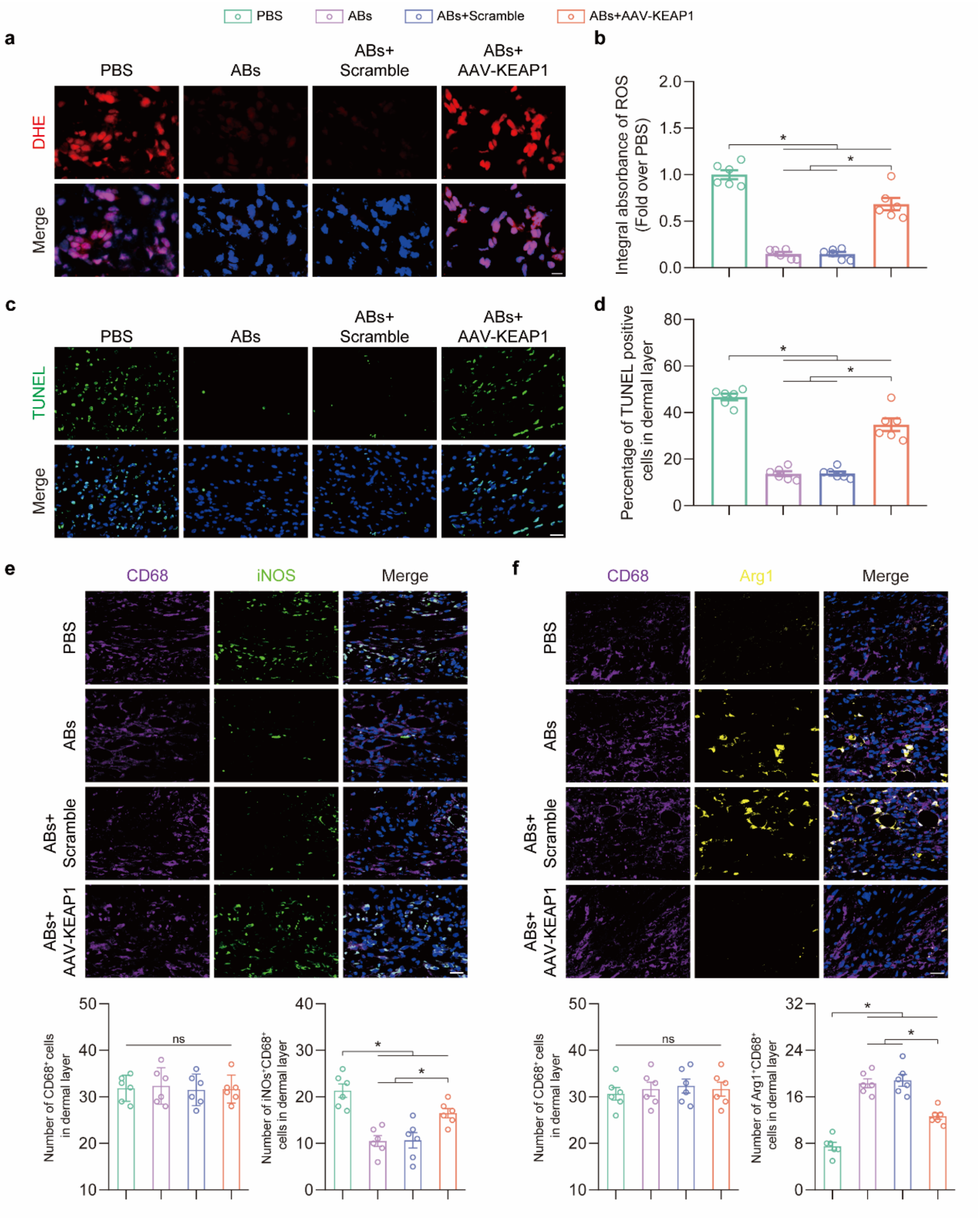

Figure S11 ABs inhibited oxidative stress and cell death and promoted the M1-to-M2 transition in ischaemic flaps by inhibiting KEAP1.

a Frozen skin tissue sections in the 4 groups on POD7 were stained with DHE. Scale bar: 10 μm. **b** Quantification of DHE in the 4 groups (n=6). **c** Dead cells in flap tissue sections on POD7 were examined by TUNEL staining. Scale bar: 20 μm. **d** Quantification of TUNEL-positive cells in the dermal layer in the 4 groups (n=6). **e**

CD68 and iNOS staining in area II of the flap in the 4 groups on POD7. Scale bar: 20 μm. The levels of infiltrated $CD68^+$ macrophages and M1-like ($CD68^+$ and $iNOS^+$) macrophages were quantified in the 4 groups (n=6). **f** CD68 and Arg1 staining of CD68 in area II of the flap in the 4 groups on POD7. Scale bar: 20 μm. The levels of infiltrated $CD68^+$ macrophages and M2-like ($CD68^+$ and Arg^+) macrophages were quantified in the 4 groups (n=6). The error bars are the SEMs. Significance (*): *p value* < 0.05; ns, not significant; ANOVA plus LSD post hoc analysis (equal variances) or Dunnett's T3 test (unequal variances).

Figure S12

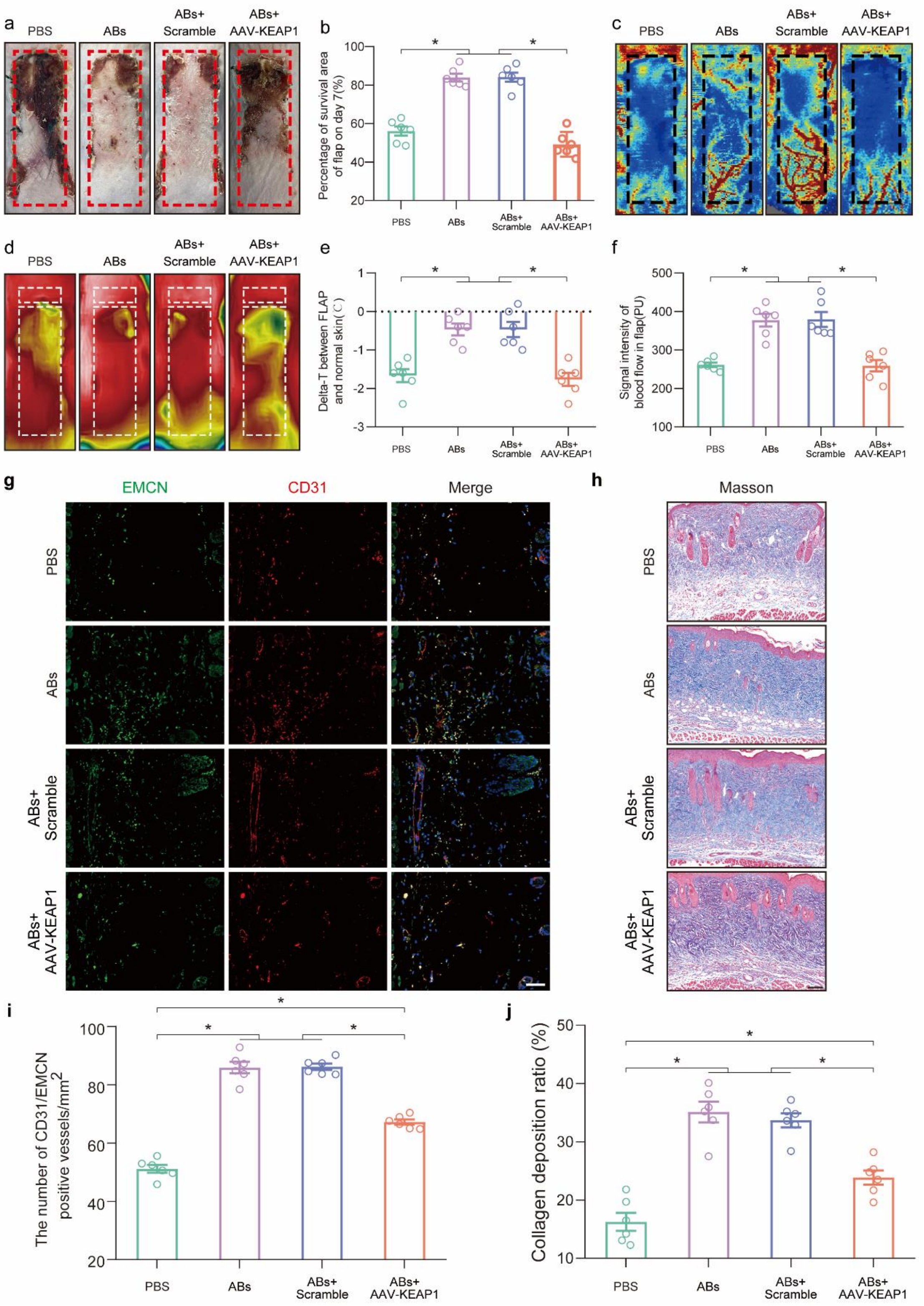

Figure S12 ABs promoted the survival of ischaemic flaps by inhibiting KEAP1.

a Digital image of the flap survival area on POD7. **b** Quantified percentage of survival area in the 4 groups on POD7 (n = 6). **c** Images of the subcutaneous blood flow network

on POD7. **d** Thermal images of the flap on POD7. **e** Comparison of the delta-T values between FLAP and normal skin in the 4 groups on POD7. **f** Quantification of the blood flow signal intensity in ischaemic flaps in the 4 groups on POD7 (n = 6). **g** IF staining of CD31 and EMCN in area II of the FLAP on POD7. Scale bar: 50 μm. **h** Quantified CD31/EMCN-positive blood vessel density in the 4 groups (n=6). **i** Masson staining to examine damaged collagen in the skin on POD7. Scale bar: 100 μm. **j** Quantification of collagen deposition ratio in the 4 groups (n=6). The error bars are the SEMs. Significance (*): *p value* < 0.05; ANOVA plus LSD post hoc analysis (equal variances) or Dunnett's T3 method (unequal variances).

Figure S13

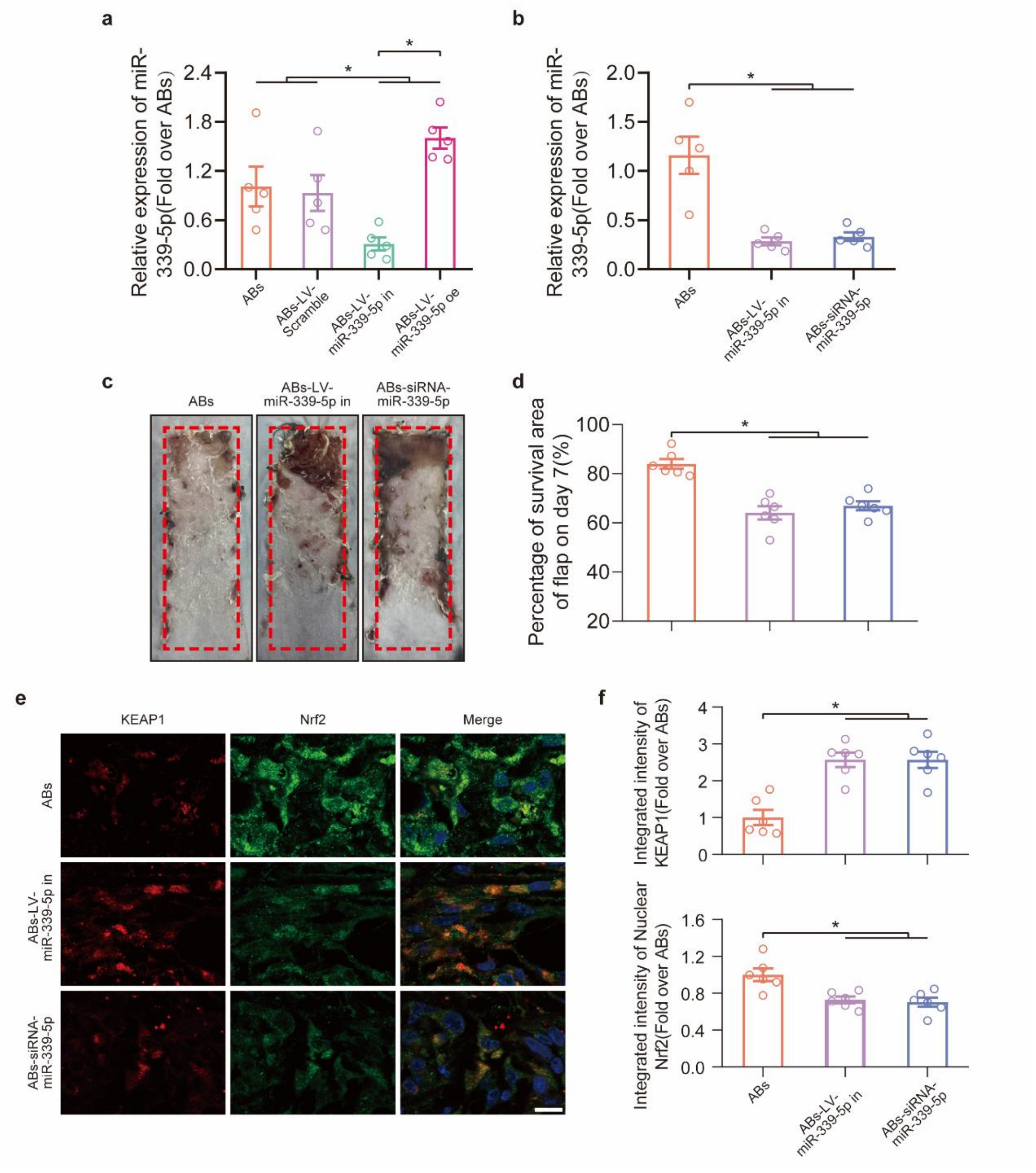

Figure S13 ABs inhibited KEAP1 and promoted flap survival by delivering miR-339-5p.

a Expression levels of miR-339-5p in the 4 groups. **b** Expression levels of miR-339-5p in the 3 groups. **c** Digital image of the flap survival area on POD7. **d** Quantification of the percentage survival area in the 3 groups on POD7 (n = 6). **e** IF staining of KEAP1 and Nrf2 in FLAP area II in the 3 groups on POD7. Scale bar: 10 µm. **f** Quantification of the integrated intensity of KEAP1 (**top**) and nuclear Nrf2 (**bottom**) in the dermal layer in the 3 groups (n = 6). The error bars are the SEMs. Significance (*): *p value* <

0.05; ANOVA plus LSD post hoc analysis (equal variances) or Dunnett's T3 method (unequal variances).

Figure S14

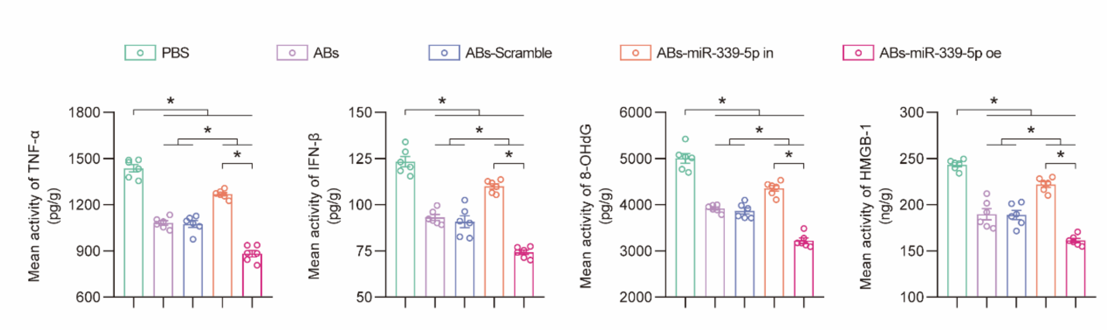

Figure S14 ELISA analysis of inflammatory factor (TNF-α, IFN-β) and DAMP (8-OHdG, HMGB-1) protein levels in area II of the FLAP in the 5 groups (n = 6). The error bars are the SEMs. Significance (*): *p value* < 0.05; ANOVA plus LSD post hoc analysis (equal variances) or Dunnett's T3 method (unequal variances).

Figure S15

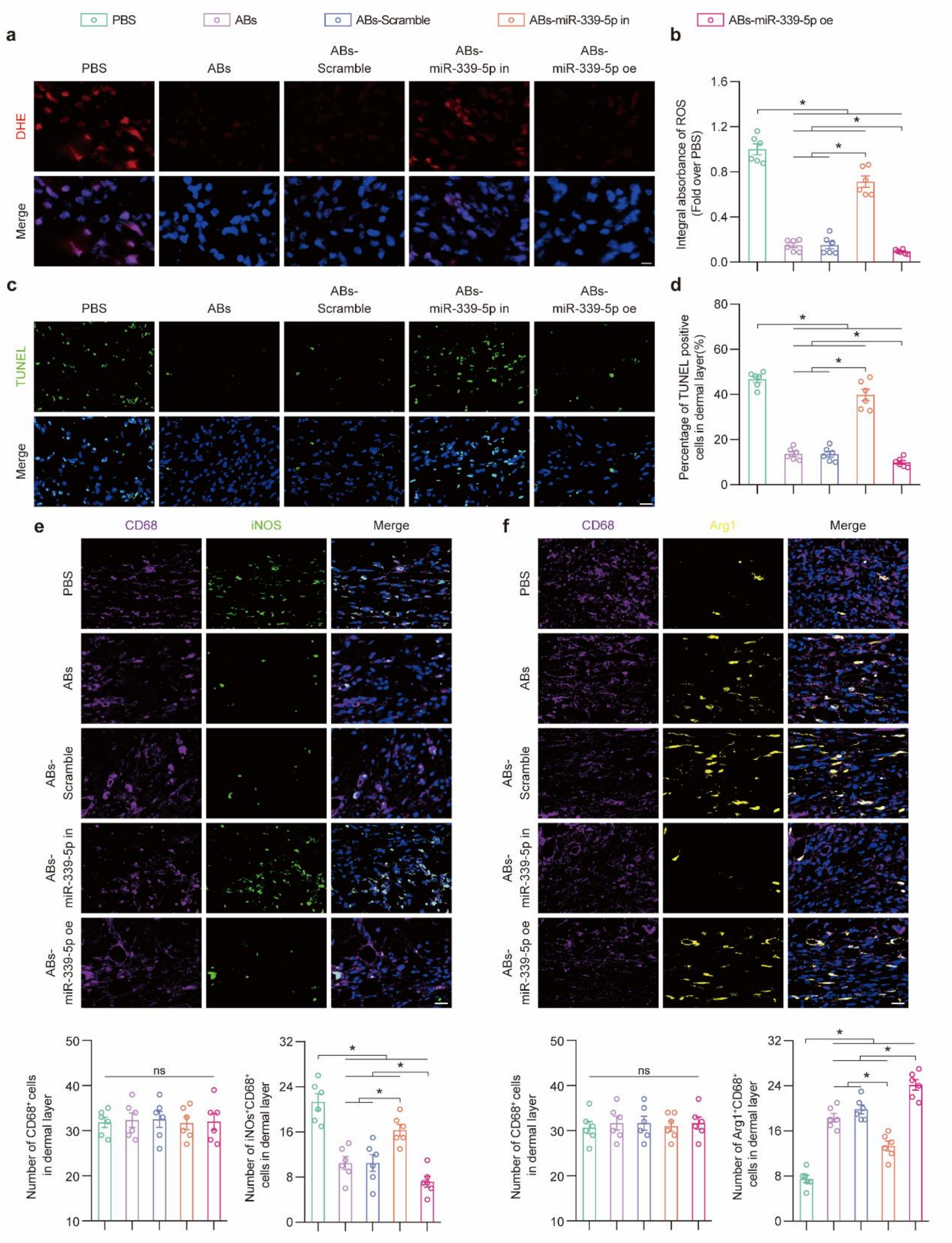

Figure S15 ABs inhibited oxidative stress and cell death and promoted the M1-to-M2 transition in ischaemic flaps by delivering miR-339-5p.

a Frozen skin tissue sections in the 5 groups on POD7 were stained with DHE. Scale bar: 10 μm. **b** Quantification of DHE in the 5 groups (n=6). **c** Dead cells in flap tissue sections on POD7 were examined by TUNEL staining. Scale bar: 20 μm. **d**

Quantification of TUNEL-positive cells in the dermal layer in the 5 groups (n=6). **e** CD68 and iNOS staining in area II of the flap in the 5 groups on POD7. Scale bar: 20 μm. The levels of infiltrated $CD68^+$ macrophages and M1-like ($CD68^+$ and $iNOS^+$) macrophages were quantified in the 5 groups (n=6). **f** CD68 and Arg1 staining of CD68 in area II of the flap in the 5 groups on POD7. Scale bar: 20 μm. The levels of infiltrated $CD68^+$ macrophages and M2-like ($CD68^+$ and Arg^+) macrophages were quantified in the 5 groups (n=6). The error bars are the SEMs. Significance (*): *p value* < 0.05; ns, not significant; ANOVA plus LSD post hoc analysis (equal variances) or Dunnett's T3 test (unequal variances).

Figure S16

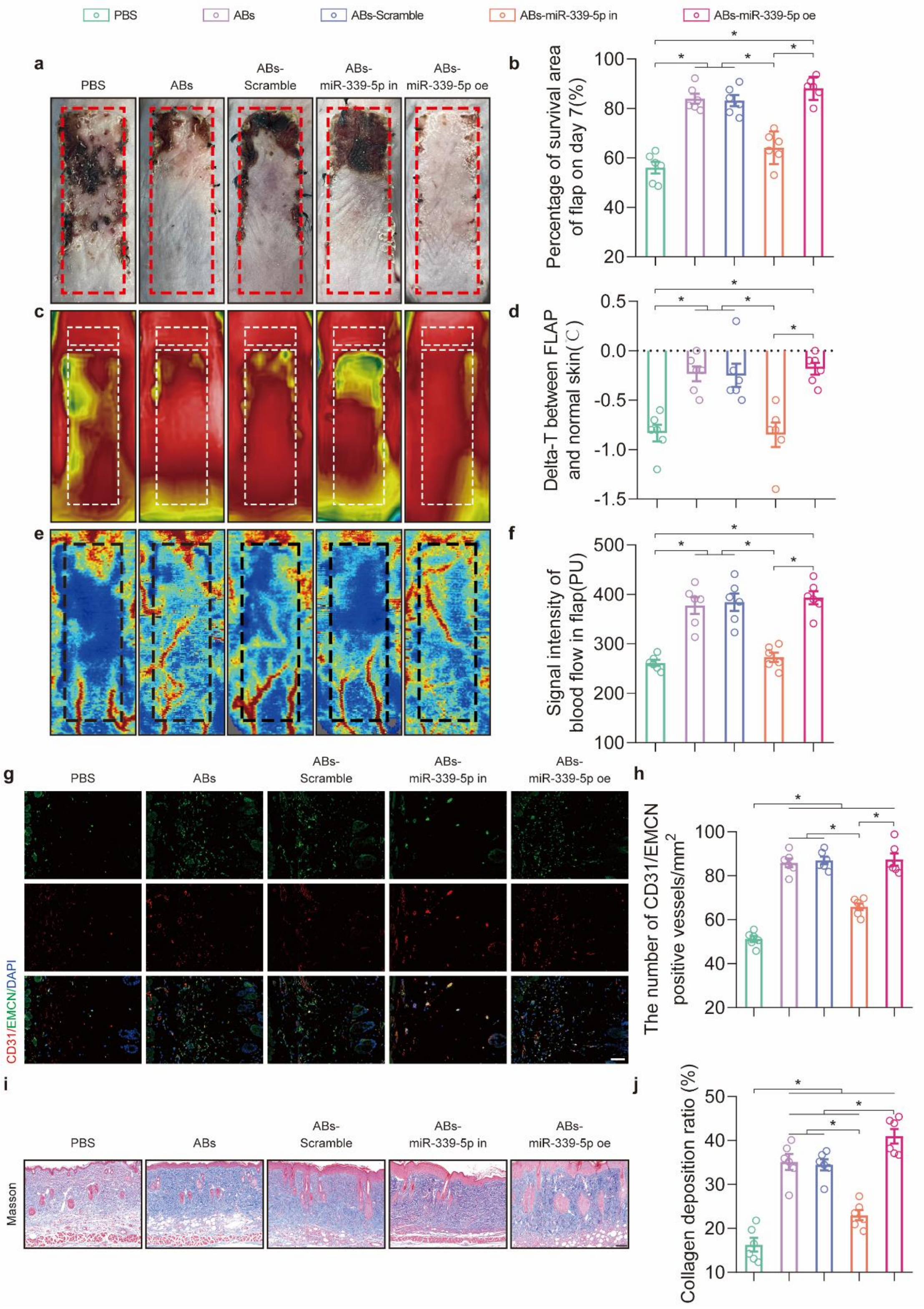

Figure S16 ABs promoted the survival of ischaemic flaps by delivering miR-339-5p.

a Digital image of the flap survival area on POD7. **b** Quantified percentage of survival area in the 5 groups on POD7 (n = 6). **c** Thermal images of the flap on POD7. **d** Comparison of the delta-T values between FLAP and normal skin in the 5 groups on POD7 (n = 6). **e** Images of the subcutaneous blood flow network on POD7. **f** Quantification of the blood flow signal intensity in ischaemic flaps in the 5 groups on POD7 (n = 6). **g** IF staining of CD31 and EMCN in area II of the FLAP on POD7. Scale bar: 50 μm. h Quantification of CD31/EMCN-positive blood vessel density in the 5 groups (n=6). **i** Masson staining to examine damaged collagen in the skin on POD7. Scale bar: 100 μm. **j** Quantification of collagen deposition ratio in the 5 groups (n=6). The error bars are the SEMs. Significance (*): *p value* < 0.05; ANOVA plus LSD post hoc analysis (equal variances) or Dunnett's T3 method (unequal variances).

Figure S17

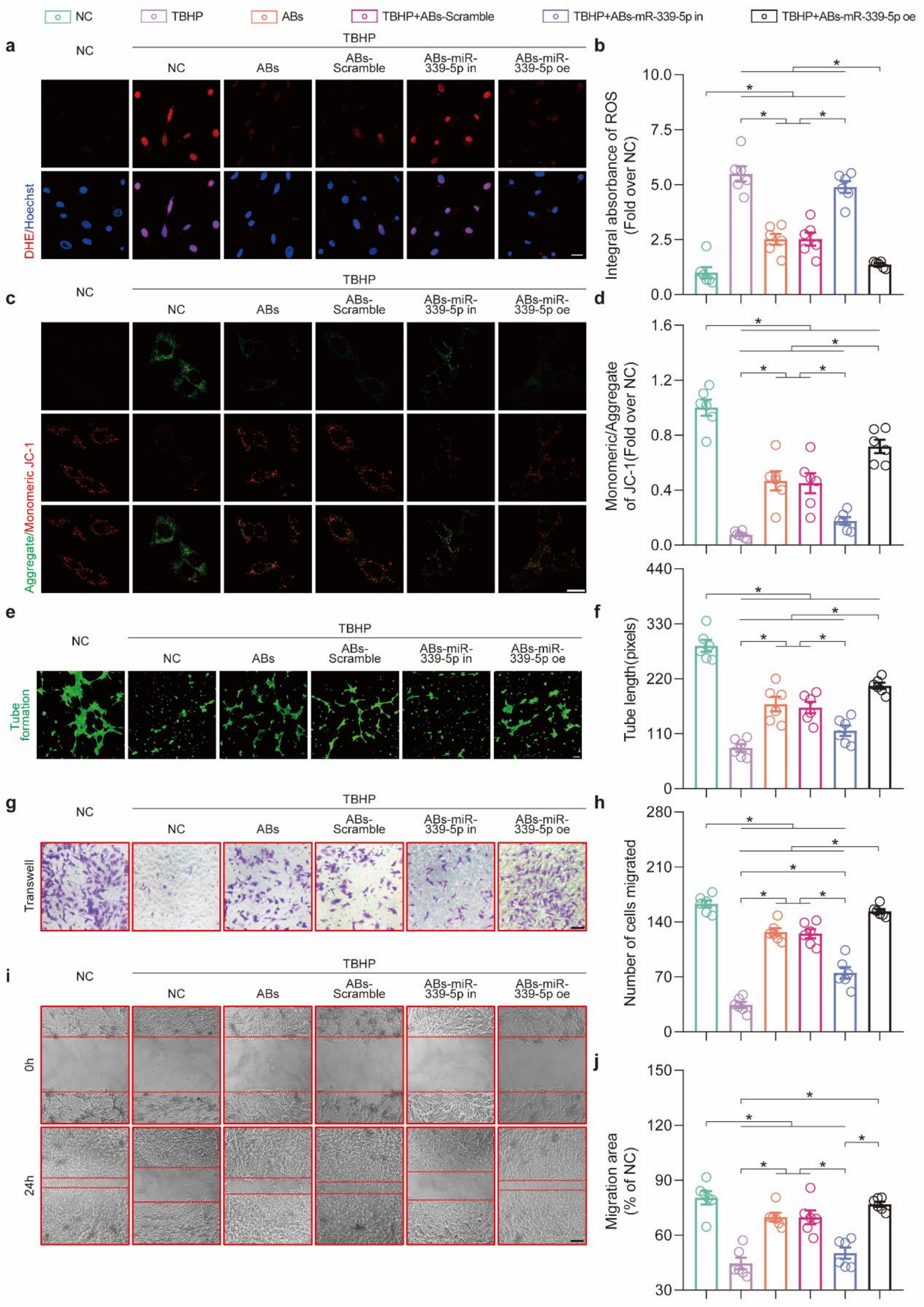

Figure S17 ABs promoted endothelial cell activity and inhibited oxidative stress by delivering miR-339-5p in *vitro*.

a DHE staining was used to examine ROS-induced DNA damage in bEnd.3 cells in the 6 groups. Scale bars: 20 μm. **b** Quantification and analysis of the integral absorbance of DHE in the 6 groups (n = 6). **c** Quantification of the ratio of monomeric/aggregated JC-1 in the 6 groups after treatment for 24 h (n=6). **d** Representative images of JC-1 staining in bEnd.3 cells in the 6 groups after treatment for 24 h. Scale bars: 20 μm. **e** bEnd.3 cells in the 6 groups were subjected to an in *vitro* angiogenesis (tube formation) assay after treatment for 24 h, and the results yielded 6 h of culture. Scale bars: 100 μm. **f** Quantification of tube length (pixels) in the 6 groups (n = 6). **g** Cell migration assays were performed on bEnd.3 cells in the 6 groups after 24 h. The results were obtained after 12 h of culture. Scale bars: 50 μm. **h** Quantification and analysis of the number of migrated cells (bEnd.3 cells) (n = 6). **i** Cell scratch experiments were performed on confluent bEnd.3 cells in the 6 groups, and measurements were performed at 0 h and 24 h. Scale bars: 500 μm. **j** Quantification and analysis of the migration area of cells after 24 h in the 6 groups (n = 6). The error bars are the SEMs. Significance (*): *p value* < 0.05; ANOVA with LSD post hoc analysis (equal variances) or Dunnett's T3 method (unequal variances).
